# Endometrial Assembloid Model Reveals Endometrial Gland Development Regulation by Estradiol‐Driven *WNT7B* Suppression

**DOI:** 10.1002/advs.202509664

**Published:** 2025-12-22

**Authors:** Xintong Li, Yanjie Guo, Jianlin Li, Yimeng Li, Leqian Lin, Hoi Kit Matthew Leung, Qingqing Zhang, Kai‐Fai Lee, Ka‐Wang Cheung, Ernest H. Y. Ng, William S. B. Yeung, Philip C. N. Chiu, Cheuk‐Lun Lee

**Affiliations:** ^1^ Department of Obstetrics and Gynaecology The University of Hong Kong Hong Kong SAR China; ^2^ Department of Health Technology and Informatics The Hong Kong Polytechnic University Hong Kong SAR China; ^3^ Shenzhen Key Laboratory of Fertility Regulation The University of Hong Kong – Shenzhen Hospital Shenzhen China; ^4^ School of Biomedical Sciences The University of Hong Kong Hong Kong SAR China

**Keywords:** assembloid, 3D culture model, endometrium, gland, organoid

## Abstract

Adult endometrial glands undergo cyclic regeneration and development during the menstrual cycle. Their secretions are vital for endometrial functions and early pregnancy, yet the mechanisms controlling gland development are not well understood. Although various 3D endometrial models exist, none fully replicate human gland development in vitro. This study establishes a robust 3D endometrial assembloid model by integrating human endometrial organoids (EOs) and human endometrial stromal cells (HESCs), successfully replicating tubular gland formation and illustrating essential stromal‐epithelial interactions. Transcriptomic analyses identify Wnt Family Member 7B (WNT7B) as an intrinsic inhibitor of gland formation and development, regulated extrinsically by transforming growth factor beta‐1 (TGFβ1) signaling through vitamin D receptor (VDR) interactions between EOs and HESCs. Endometrium‐specific WNT7B knockout mice exhibit enhanced gland development further supports WNT7B's inhibitory role in endometrial gland development. Estradiol facilitates tubular gland formation by suppressing WNT7B expression in vitro, which is confirmed in estradiol‐stimulated mouse models and clinical samples from women undergoing ovarian stimulation for in vitro fertilization. These findings elucidate the central roles of estradiol‐WNT7B signaling and stromal‐derived TGFβ1‐VDR crosstalk in endometrial gland development, providing a foundation for improved 3D endometrial models and identifying therapeutic targets for gland‐related disorders like endometriosis, infertility, and endometrial hyperplasia.

## Graphical abstract

This study developed a 3D endometrial assembloid model to study how uterine glands form and develop. They discovered key interactions between different cell types and identified WNT7B as a regulator controlled by estradiol‐mediated TGFβ1‐VDR interaction. These findings confirmed in in vitro, animal and clinical studies, provide a foundation for advanced 3D models and potential treatments targeting disorders related to uterine glands.

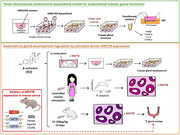

## 1 Introduction

Endometrial glands perform secretory functions during the mid‐secretory phase of menstrual cycle and early pregnancy [1, 2, 3]. Endometrial gland development in adults involves cyclical remodeling, elongation, branching, and regeneration of existing glands, driven by ovarian hormones and interactions between stromal and epithelial cells. Unlike de novo postnatal adenogenesis, this process modifies preexisting glandular structures. During each menstrual cycle, estradiol promotes epithelial proliferation and differentiation, shaping glandular architecture and enhancing secretory function in the proliferative phase. This cyclical glandular regeneration is crucial for preparing the uterus to support embryo implantation and development [4, 5]. Despite their fundamental importance in reproductive physiology, the mechanisms governing the morphogenesis and development of human endometrial glands remain poorly understood.

In recent years, various in vitro endometrial models have been created to overcome the challenges of studying human endometrium in vivo [6, 7, 8, 9, 10, 11]. 3D endometrial models have been developed by co‐culturing of endometrial epithelial and stromal cells using Collagen I or Matrigel as an extracellular matrix (ECM) scaffold [12]. These 3D endometrial models offer advantages over conventional 2D models, which exhibit diminished biological activities during long‐term cultures. In contrast, 3D models better preserve cellular structure, physiological properties, and functionality [13]. Building upon these 3D modeling approaches, endometrial organoids (EOs) are innovative models that effectively mimic the morphology and functions of human endometrial glands. These organoids are self‐organizing and genetically stable for long term [6, 14, 15, 16]. Derived from primary endometrial tissue, EO contains various cell types such as ciliated cells, proliferative cells, secretory cells, and stem cells [17]. Recently, researchers have advanced these models by generating endometrial assembloids through the integration of EOs with other endometrial cell types such as stromal cells [18], creating systems that more closely simulate in vivo endometrial conditions. However, even these sophisticated models cannot fully replicate the development of epithelial glands of endometrium [2, 19]. In general, the mechanism of gland development is broadly conserved across organs including salivary glands, intestinal glands, mammary glands [20]. The specific molecular pathways governing human endometrial gland development remain elusive. Both intrinsic signaling, such as the Wingless‐related integration site (Wnt) pathway, and extrinsic factors from the surrounding stroma regulate endometrial gland development [2, 21, 22, 23, 24]. Although researchers have identified molecular drivers and inducible factors for adenogenesis in salivary and mammary gland organoids [25, 26], comparable knowledge for human endometrial glands is lacking.

Hormonal regulation by β‐estradiol provides physiological dimension to endometrial gland development due to its regulatory functions throughout the menstrual cycle [27]. During the proliferative phase, rising estradiol levels stimulate growth and proliferation of endometrial glands, which are crucial for the cyclic development of the endometrium [28]. At the molecular level, estrogen receptors (ERs) interact with multiple growth factors and signaling pathways, particularly the Wnt and Transforming Growth Factor beta‐1 (TGFβ1) pathways to regulate estradiol‐induced growth and development of the endometrial glands [29, 30]. From a clinical perspective, disorders related to abnormal endometrial gland development, such as endometrial hyperplasia, are associated with elevated systemic or local estradiol levels through mechanisms that warrant further investigation [31, 32].

Integrating these structural, molecular, and hormonal perspectives, this study established a robust 3D endometrial assembloid model combining EOs and human endometrial stromal cells (HESCs) to investigate gland development in vitro. The established assembloid model spontaneously self‐organizes into lumenized tubular glands within a 3D stromal matrix, recapitulating the structural hierarchy of human endometrial glands. This model reveals the crucial role of *WNT7B* in regulating human endometrial gland development. The findings are validated through complementary experimental approaches including an endometrial‐specific *WNT7B* knockout mouse model, an estradiol‐stimulated mouse model, and analyses of human endometrium obtained from women undergoing controlled ovarian stimulation during in vitro fertilization (IVF) treatment.

## 2 Methods

### 2.1 Cell Culture

Telomerase‐transformed human endometrial stromal cell lines (T HESCs, ATCC CRL‐4003, RRID:CVCL_C464, acquired in Mar 2020) were purchased for use in the study and cultured with 1:1 mixture of DMEM/F‐12 medium with 3.1 g L^−1^ glucose and 1 mM sodium pyruvate and without phenol red (D2906, Sigma) supplemented with 1% Penicillin/Streptomycin (Thermo Fisher Scientific), 1.5 g L^−1^ of sodium bicarbonate (Sigma, USA), 1% ITS+ Premix (BD Biosciences), and 10% charcoal/dextran treated fetal bovine serum (FBS; HyClone). The medium was changed every 2 days and the cells were passaged at around 80%–90% confluency. The cells were confirmed to be mycoplasma free by the HKU Bioresearch Support Core.

### 2.2 Derivation and Culture of Endometrial Organoid

The protocol of this study was approved by the Institutional Review Board (IRB UW 19‐210, UW 20‐060 & UW 21‐970) at The University of Hong Kong/Hospital Authority Hong Kong West Cluster. Human endometrial tissue was procured through endometrial biopsies of infertile women undergoing IVF treatment with written consent. Endometrial organoid (EOs) were established and cultured according to previously published procedure [33, 34]. Briefly, the endometrial tissue was washed with Advanced DMEM/F12 Medium (Life Technology) and cut into small pieces at around 1 mm^2^, then digested with the enzyme solution (0.4 mg mL^−1^ Collagenase type V (Sigma), DNase I (Roche)) at 37 °C for 10–15 min. The endometrial glandular elements were collected by filtering through 100 µm cell strainers (Thermo Fisher Scientific) and subsequent backwashing. The obtained glandular cells were embedded in 20 µl Matrigel (Corning) at a ratio of 1:10 (v:v), solidified at 37 °C for 20 min, then filled with 200 µl of the EO Expansion Medium (ExM). For EO passaging, EO‐containing Matrigel was scraped and collected in basal medium and centrifuged at 1000 rcf for 5 min. The cell pellet was resuspended in 500 µl of basal medium and pipetted up and down for 100 times, followed by centrifugation at 1000 rcf for 5 min. The cell pellet was resuspended in ice‐cold Matrigel and released as 20 µl‐droplets in a 48‐well plate with 200 µl of ExM. The EOs were obtained between 2019‐2023 and were confirmed to be mycoplasma free by the HKU Bioresearch Support Core.

### 2.3 3D Assembloid Model Establishment

EOs were washed in basal medium and collected into 200 µl of cell recovery solution (Corning) per well, left on ice for 30 min with several vortex, then centrifuged at 1000 rcf for 5 min. HESCs were trypsinized and centrifuged at 500 rcf for 5 min. The pellets of the EOs and the HESCs were mixed and resuspended into the culture medium supplemented with 90% ExM and 10% Matrigel for hanging‐drop co‐culture, at a concentration of 5 × 10^3^ HESCs and 2–3 EOs per 10 µl of ExM/Matrigel. The cell mixture was seeded onto the lid of a 24‐well culture plate with two 10 µl‐drops per well and the plate lid was inverted. The lower wells of the culture plate were filled with sterile PBS (Sigma) to prevent drying. The cells were cultured at 37 °C for 48 h for assembloid formation. The co‐culture model was established after the 48 h hanging‐drop co‐culture. HESCs were trypsinized and centrifuged at 500 rcf for 5 min, 1.5 × 10^5^ of HESCs were resuspended in 250 µl of 80% Collagen (50% Collagen I (Corning), 7.5% 20X PBS, 40% water, 2.5% 1 M sodium hydroxide)‐20% Matrigel‐hydrogel complex, then seeded into the Nunc 4‐well culture plate (Thermo Fisher Scientific). The EO‐HESC‐assembloids were immediately transferred into the Collagen‐Matrigel‐hydrogel complex manually under a dissection microscope to allow 5‐10 assembloids contained in each well. The co‐culture model was incubated at 37 °C in incubator for 1 h to solidify. ExM was added for the first 24 h, then changed to complete growth medium of HESCs. The medium was changed every 2 days. Bright‐field images were captured under an inverted microscope (Nikon TE300). Tubular lengths were measured once a day for 5 days after the tubular structure is formed using the inbuilt SPOT Software 5.0 (Diagnostic Instruments, Inc.).

### 2.4 Conditioned Medium Collection and Concentration

Conditioned medium from the assembloid model was collected separately on day 1 or day 7 of co‐culture. On day 1 and day 7, the original culture medium was removed. The co‐culture model was washed with DMEM blank media (phenol‐red free without FBS, D2906, Sigma) for four times, followed by addition of 400 µl of fresh DMEM blank medium. The conditioned medium was collected after 24 h, followed by centrifugation at 500 rcf for 3 min to remove any remaining cells in the medium. Amicon Ultra‐0.5 10 K Centrifugal Filter Device (Millipore) was used to concentrate the conditioned medium according to manufacturer's protocol. Pierce BCA protein assay kit (Thermo Fisher) was used for protein amount determination in the concentrated conditioned medium.

### 2.5 Proteomics Analysis

The concentrated conditioned medium was sent for proteomics analysis in Beijing Genomic Institute (BGI), Hong Kong. Quantitative protein identification was conducted using the Isobaric Tags for Relative and Absolute Quantitation (iTRAQ) technology according to a published protocol [33]. Proteins with mean fold change greater than 1.2 and p value lower than 0.05 were selected as DEPs.

### 2.6 Trophoblast Stem Cell (TSC) Culture and Extravillous Trophoblast (EVT) Differentiation

Human TSC lines were derived from human expanded potential stem cells (hEPSCs), cultured and differentiated according to published protocol [35]. In brief, six‐well plate was pre‐coated with 1% Geltrex (Thermo Fisher) at 37 °C for 1 h. TSCs were seeded at a density of 2 × 10^5^ cells per well in 2 mL of TSC medium in 37 °C, 5% CO_2_ incubator, and the culture medium was changed every 2 days. When cells reach 80% confluency, TSCs were digested with 1 mL of TrypLE (Thermo Fisher) and subcultured at the ratio of 1:5. For EVT differentiation, TSCs were digested with 1 mL of TrypLE, then seeded in 1% Matrigel‐coated 6‐well plates at a density of 1 × 10^5^ cells in 2 mL of Day 0 differentiation medium. The medium was changed to day 3/day 6 differentiation medium on respective days. The derived TSCs were confirmed to be mycoplasma free by the HKU Bioresearch support Core.

### 2.7 Transwell Invasion Assay

EVTs were differentiated in vitro from TSCs until day 6 for transwell invasion assay according to previously published protocol [36, 37]. EVTs were digested by TrypLE and seeded in the upper chamber of rehydrated invasion chamber at a density of 1.2 × 10^5^ cells in 300 µl of basal blank EVT medium. Thirty micrograms of day 1 or day 7 conditioned medium from assembloids or blank medium at the same volume were added to the upper chamber separately. The lower chamber of the invasion chambers was loaded with 650 µl of 20% FBS. Cells were incubated at 37 °C for 20 h, followed by gentle removal of cells in the upper chamber by swiping using cotton sticks. The invasion chamber was stained in 0.5% Crystal Violet (Sigma) for 15 min, then washed in distilled water for 2 times and dried. Five fields were randomly selected and imaged at 10x magnification for each reaction, and the mean number of invaded cells was quantified by the Image J software (US National Institutes of Health) for calculation of relative invasion ratio.

### 2.8 Bulk Transcriptomes

EOs used for co‐culture model establishment were divided into two categories, namely those with and without tubular gland development capacity. EOs with (*n* = 2) and without (*n* = 2) tubular gland development were collected into 900 µl of the RNAiso Plus solution (TaKaRa) for RNA extraction. The RNA samples were proceeded for DNBSEQ Eukaryotic Strand‐specific Transcriptome Resequencing with Paired‐end (PE)‐150 in BGI, Hong Kong. For transcriptome analysis, quality control was conducted with *fastQC* (0.11.8) and *fastp* (0.20.0) [38]. Reads passed the quality control procedure were aligned to the human reference genome hg38 by *Hisat2* (2.1.0) [39]. Gene counts were calculated via *featureCounts* (1.6.4) [40], and the gene expression level read count matrix was analyzed by R studio (4.0.3) via three different plugin packages, including *DEseq2* (1.30.0) [41], *edgeR* (3.32.0) [42] and *limma* (3.46.0) [43]. DEGs with *p* value ≤ 0.05 and fold change ≥ 2/≤ 0.5 were selected and intersected from the database obtained using the three packages, given as DEGs for further analysis. Heat map was generated by *pheatmap* (1.0.12). Enriched GO analysis was generated by *clusterProfiler* (3.18.1) [44].

### 2.9 Single‐Cell RNA Sequencing Analysis

The 3D EO/HESC co‐culture model on day 1 and day 9 after establishment were digested in 300 µl of Collagenase (1 mg mL^−1^) for 30 min at 37°C, followed by incubation in 500 µl of Trypsin‐EDTA and 50 µl of DNase for 5 min at 37 °C with shaking at 1000 rpm. The reaction was stopped by 50 µl of 100% FBS. The cells were washed in PBS and filtered through a 40 µm cell strainer for the removal of cell clumps. The so‐obtained single‐cell suspension was resuspended in 4% Bovine Serum Albumin (BSA, Sigma) and sent for single‐cell RNA sequencing. Library preparation and Illumina sequencing were conducted at the Genomics Core, Centre for PanorOmic Sciences (CPOS), LKS Faculty of Medicine, the University of Hong Kong. Raw data was first processed with *Cell Ranger* (6.0.0) [45]. Downstream analysis was conducted using the *Seurat* (4.1.0) [46] package in R studio, including normalization, shared nearest neighbour graph‐based clustering, differential expression analysis and visualization, with the pipeline suggested as standard practice [47]. Input data from day 1 co‐culture model and day 9 co‐culture model was normalized and integrated for Uniform manifold approximation and projection (UMAP) analysis and cluster identification. Clusters were identified using the ‘FindClusters’ function from the Seurat package. UMAP plot was generated using the ‘RunUMAP’ function with default parameters. Violin plot was generated using the ‘VlnPlot’ function. DEGs with raw *p* value ≤ 0.05 and fold change ≥ 2/≤ 0.5 were selected. Enriched GO analysis for biological function was generated by clusterProfiler.

Single‐cell RNA sequencing analysis data of full‐thickness human endometrium was downloaded from the article published by Garcia‐Alonso and co‐workers [48]. The data was imported into R studio, normalized and integrated to this single‐cell RNA sequencing analysis data in the Seurat package. *CellPhone DB* (4.1.0) [49] was used for analysis of cell‐cell communication from single‐cell RNA sequencing analysis data. Ligand‐receptor pairs were identified by ‘statistical analysis’ functions with default parameters.

### 2.10 Establishment of *WNT7B* Knockdown EO Model by CRISPR Cas9

#### 2.10.1 Lentivirus Preparation

Two single‐guidance RNA (sgRNA) oligos targeting *WNT7B* exon‐2 were designed using the SnapGene software 6.0.2 (Dotmatics): sgRNA1‐Forward: CCCGATGCCATCATTGTGAT; sgRNA1‐Reverse: ATCACAATGATGGCATCGGG; sgRNA2‐Forward: GAAGACGGTCTTCTCGCCGA; sgRNA2‐Reverse: TCGGCGAGAAGACCGTCTTC. Lenti‐*WNT7B* sgRNA‐Cas9 lentivirus was synthesized using the GV708 vector (U6‐sgRNA‐EF1a‐Cas9‐FLAG‐CMV‐EGFP‐P2A‐puro), and the two sets of sgRNAs were constructed into plasmid separately. The negative control group was inserted with a non‐targeting oligo (CGCTTCCGCGGCCCGTTCAA) with the same plasmid vector, named as lenti‐GFP‐Cas9 plasmid. The work of plasmid construction and lentivirus synthesis was conducted by the Gene Company, China. The final titer of lenti‐*WNT7B* sgRNA1‐Cas9, lenti‐*WNT7B* sgRNA2‐Cas9 and lenti‐GFP‐Cas9 plasmids were prepared at 2 × 10^8^ infectious units/ml.

#### 2.10.2 Lentivirus Transduction

One confluent well of EOs were seeded into each well of 24‐well plate in ExM without Matrigel, allowing them to attach on the bottom of the well. Non‐attached EOs were removed on the second day, and lentivirus transduction was conducted on day 4. The transduction system was prepared with 400 µl of ExM, 3 µl of ViraDuctin Reagent A (Cell Biolabs), 3 µl of ViraDuctin Reagent B, and equal volumes of lenti‐*WNT7B* sgRNA1‐Cas9 and lenti‐*WNT7B* sgRNA2‐Cas9 achieving a multiplicity of infection (MOI) = 10. The negative control group was prepared with lenti‐GFP‐Cas9 plasmid at MOI = 10 together with same volume of ExM and ViraDuctin reagents. The reaction system was mixed and incubated at 37 °C for 30 min. The original ExM in the cultured EO cells was then replaced with the *WNT7B*‐targeting lentivirus mixture or GFP lentivirus mixture. The EO cells were infected for 16 h at 37 °C. On day 5, the infection reaction was stopped by removal of lentivirus mixture and washing in ViraDuctin Reagent C prepared in ExM for 60 s with gentle shaking. The EO cells were then washed in basal medium for 2 times and incubated in fresh ExM overnight.

#### 2.10.3 Drug Selection

On day 6, successfully transfected EO cells were selected by Puromycin treatment at a concentration of 0.6 µg mL^−1^, which was tested as the optimal concentration that killed 90% of the wildtype EO cells in 72 h. Fluorescence signal of remaining cells was checked under a fluorescence microscope (Nikon) 72 h after drug selection. Drug selection was stopped by removal of Puromycin and washing of EO cells in basal medium, then replaced with fresh ExM.

#### 2.10.4 EO Reformation and Tube Formation

After 24 h of incubation in ExM, the transfected EO cells were digested by TrypLE and centrifuged at 1000 rcf for 5 min, then seeded in 20 µl of Matrigel and incubated in ExM. Images of the EOs were captured at the excitation wavelength of 488 nm under a fluorescence microscopy and Carl Zeiss LSM 800 inverted confocal microscope under Zeiss LSM Zen 2010 software (Carl Zeiss) at CPOS, The University of Hong Kong. Co‐culture model was established using *WNT7B* knockdown EOs according to previous protocol. Five to ten assembloids formed from transfected EOs were transferred into each co‐culture model. Proportion of EOs with tube development was calculated by the number of EOs with tube development on day 10 divided by the number of EOs per model.

### 2.11 Interaction between VDR and TGFβ1

Co‐immunoprecipitation was conducted as described [36, 50] to confirm the interaction between VDR and TGFβ1. Briefly, 5 µg of recombinant VDR (12025‐H08B, Sino Biological) or 500 µg of EO protein lysate was incubated with 5 µl of monoclonal anti‐VDR (ab3508, Abcam) or isotype antibody overnight at 4 °C. Dynabeads Protein G (Thermo Fisher Scientific) were added to capture VDR and its interacting partners. The beads were then washed, and the bound protein complex was eluted and analyzed by Western blot using antibodies against VDR (ab3508, Abcam) and TGFβ1 (ab215715, Abcam).

### 2.12 TGFβ1 Treatment in EOs

Recombinant human TGFβ1 protein (R&D systems, 240‐B) was reconstituted at 20 µg mL^−1^ in sterile 4 mM HCl containing 1 mg mL^−1^ of BSA, then prepared at a working concentration at 10 ng mL^−1^ in ExM. EOs were subcultured and grown in ExM for 3 days, then replaced with 10 ng mL^−1^ of TGFβ1 and incubated for 48 h. The control group was replaced with fresh ExM. After 48 h, the medium was removed and EOs were collected for RT‐qPCR or co‐culture model establishment. For tube development assessment, length of tubular structure developed from the EOs was measured on day 10 after co‐culture using SPOT software 5.0.

To examine the effect of VDR inhibition, EOs were treated with 1 µM of ZK168281 (MCE), 1 µM of TEI‐9647 (MCE) or 1 µM of TEI‐9648 (MCE) and with 10 ng mL^−1^ of TGFβ1 for 48 h. EOs were collected for RT‐qPCR analysis for WNT7B expression examination.

### 2.13 Estradiol Treatment in EOs

β‐Estradiol (Sigma–Aldrich, E4389) was reconstituted in sterile PBS, and prepared at a working concentration at 10 or 100 nM in ExM. EOs were subcultured and grown in ExM for 3 days, then replaced with 10 or 100 nM of estradiol and incubated for 7 days. The estradiol‐containing medium was changed every 2 days. After 7 days, the medium was removed and EOs were collected for immunohistochemical staining, RNA extraction or assembloid formation and subsequent co‐culture model establishment. The co‐culture model was incubated in estradiol‐containing culture medium at 10 nM (E2/ExM 10 nM + E2/DMEM 10 nM) or 100 nM (E2/ExM 100 nM + E2/DMEM 100 nM), respectively. The control groups were established with EOs without estradiol pretreatment and without estradiol supplementation in the co‐culture (WT + DMEM), EOs without estradiol pretreatment and with 10 nM estradiol supplementation in the co‐culture (WT + E2/DMEM 10 nM), or EOs without estradiol pretreatment and with 100 nM estradiol supplementation in the co‐culture (WT + E2/DMEM 100 nM). Proportion of EOs with tube development was calculated as mentioned before.

### 2.14 Generation of Estradiol‐Stimulated Mouse Model

The animal experiments were approved by the Committee on the Use of Live Animals in Teaching and Research (CULATR: 5317‐20; 5938‐21) of the University of Hong Kong. Female mice of the strain ICR1 at an age of 6‐8 weeks were used in the study, which were purchased and housed in the Centre of Comparative Medicine Research at the University of Hong Kong. Mice were caged in a pathogen‐free chamber. To maintain their circadian rhythm, mice used in the experiments were exposed to 14 h of light and 10 h of dark every day. Estrous‐cycle staging was performed by daily vaginal cytology for approximately two weeks prior to dosing. Vaginal smears were taken in the late afternoon to identify proestrus phase (day 0), and the first intraperitoneal (i.p.) injection was administered in the following morning (day 1), within 12–16 h of staging according to vaginal smear result stained in Haematoxylin for 30 min. β‐Estradiol (E4389, Sigma) was prepared in PBS at a concentration of 10 µg mL^−1^. Female mice at an age of 8 weeks were injected with estradiol at a dose of 100 µg kg^−1^ i.p. every 24 h for 14 days. The control group was injected with PBS at the same volume following the same procedure. After 14 days of injection, the mice were sacrificed by an overdose of sodium pentobarbital (150 mg kg^−1^ i.p.) followed by cervical dislocation. The uterus was collected and weighted for the calculation of uterine mass‐to‐body weight ratio. Endometrial tissue from both sides of the uterus of each mouse was collected, then fixed in 4% PFA overnight and transferred into 70% ethanol for paraffin embedding. Endometrial gland number and diameter were quantified according to H&E staining using ImageJ software. To calculate average gland number, one uterine segment was obtained from each side of the uterus, and three non‐consecutive sections were stained for each vertically‐embedded uterine segment. The average number of glands was calculated from a total of six sections from both sides of the uteri. To calculate gland diameter, ten glands were randomly selected from each section, and the average gland diameter was calculated.

### 2.15 Human Endometrial Tissue

Human endometrium tissue from women with normal menstrual cycles at proliferative and secretory phase was provided by the Department of Pathology, Queen Mary Hospital, Hong Kong, for immunohistochemical staining. All women provided written consent for tissue collection. Ethical approval was obtained from the Ethics Committee of the Faculty of Medicine, the University of Hong Kong.

### 2.16 Human Endometrial Tissue under Controlled Ovarian Stimulation

Human endometrial tissue was collected from infertile women undergoing IVF cycles in the Assisted Reproduction Unit at the Department of Obstetrics and Gynecology, Queen Mary Hospital, Hong Kong, with written consent. Ethical approval was issued by the Ethics Committee of the Faculty of Medicine, The University of Hong Kong. Endometrial tissue was collected from women using the long protocol of ovarian stimulation by pretreatment with gonadotrophin releasing hormone (GnRH) analogue, buserelin nasal spray (Suprecur; Hoechst AG, Germany), at 150 µg four times a day from the mid‐luteal phase, followed by treatment with human menopausal gonadotropin (hMG, Pergonal; Serono, Switzerland) after pituitary downregulation. hCG (Profasi; Serono, Switzerland) was then given at a dose of 10 000 IU when the leading follicle reached 18 mm and 3 or more follicles showed diameters greater than 15 mm. Serum estradiol level of the women was measured at hCG injection day and hCG+7 day. Endometrial tissue biopsy was conducted at hCG+7 day. Women in the control group recruited for the study were patients with regular menstrual cycle and male‐factor infertility. Their endometrial tissue was biopsied at LH+7 day, and their serum estradiol level was measured at LH surge day and LH+7 day. Mann‐Whitney U test for unequal variances was used to compare two independent groups.

### 2.17 Generation of *Wnt7b^f/f^; Pgr Cre/+* Mouse Model

The animal experiments in this study were approved by CULATR: 23‐246; 23‐235 in The University of Hong Kong. Female mice of the strain B6;129X1‐*Wnt7b^2Amc^*/J (#008467) [51] and male mice of the strain B6.129S(Cg)‐*Pgr^1.1(cre)Shah^*/AndJ (#017915) [52] were purchased from the Jackson Laboratory, USA. The mice were bred to generate the offsprings with the genotype of *Wnt7b^f/+^; Pgr Cre/+* or *Wnt7b^f/f^; Pgr +/+*, followed by interbreeding to generate the offsprings.

Genotyping for the mice was conducted after weaning by ear punching. DNA was extracted from the earpiece using the TIANamp Genomic DNA kit (Tiangen) according to manufacturer's protocol. PCR was conducted in 20 µl system with 10 µl of 2X Master Mix (TaKaRa), 4 µl of primer mix (oIMR7415, oIMR9384, oIMR9385 or 14565, 14566, 14567) and 6 µl of DNA at 98 °C for 10 s, 68 °C for 30 s and 72 °C for 1 min for 34 cycles. Primers oIMR7415, oIMR9384 and oIMR9385 (oIMR7415, 5’‐GCCAGAGGCCACTTGTGTAG‐3’, oIMR9384, 5’‐GGTAGTCCTTCCTGCCCTTT‐3’, oIMR9385, 5’‐GTGTGTCCTGGCCTGATTTT‐3’) were used to amplify the fragments from the wild‐type (344 bp) and floxed (221 bp) allele of *WNT7B*. Primers of 14565, 14566 and 14567 (14565, 5’‐AGTTATTGCTGCCCAGTTGC‐3’, 14566, 5’‐CCCTTCTCATGGAGATCTGTC‐3’, 14567, 5’‐GCGCTAAGGATGACTCTGGTC‐3’) were used to amplify the *Pgr* wild‐type (419 bp) and *Cre* (600 bp) alleles. Female *Wnt7b^f/f^; Pgr Cre/+* mice were identified as the conditional knockout genotype. Female *Wnt7b^f/f^; Pgr +/+* mice were identified as the wildtype control group.

The female mice of designated genotypes were obtained at 6 weeks. Vaginal smear was conducted, and the wildtype and mutant mice were sacrificed at proestrus phase. Uterine tissue was cut into sections and fixed in 4% PFA overnight, followed by paraffin embedding. Number of endometrial glands were counted as mentioned in *‘Generation of estradiol‐stimulated mouse model’* section using ImageJ software.

### 2.18 H&E Staining and Immunohistochemistry

H&E and immunohistochemical staining were conducted in paraffin sections of EOs, EO‐HESC‐assembloids, 3D co‐culture model on day 1 and day 9, mouse uterine tissue and human uterine tissue. For immunohistochemical staining, paraffin‐embedded tissue slides were dewaxed and heated for target antigen retrieval, followed by incubation in 3% H_2_O_2_ for 30 min and blocking in 10% goat serum (Dako) for 1 h. Primary antibodies against FOXA2 (1:200, ab108422, Abcam, RRID:AB_11157157), Tenascin C (1:100, ab108930, Abcam, RRID:AB_10865908), PAEP (1:100, ab17247, Abcam), MUC‐1 (1:200, ab109185, Abcam, RRID:AB_2159754), MMP‐3 (1:100, ab53015, Abcam, RRID:AB_881242), IL‐6 (1:200, ab9324, Abcam, RRID:AB_307175), WNT7B (1:200, PA5‐103480, Invitrogen, RRID:AB_2852816) and TGFβ1 (1:100, ab215715, Abcam, RRID:AB_2893156) were applied overnight at 4 °C, followed by incubation in biotinylated secondary antibodies (Goat anti‐rabbit IgG (1:500, E0432, Dako, RRID:AB_2313609), Goat anti‐mouse IgG (1:500, E0433, Dako, RRID:AB_2687905) for 1 h at room temperature. Immunodetection was performed using VECTASTATIN Elite ABC kit (Vector) and subsequent DAB (Dako) exposure. The slides were counterstained in Haematoxylin for 2 min and mounted for image capture under Zeiss Microscope.

### 2.19 Immunofluorescence

Immunofluorescence staining was conducted in paraffin sections of the co‐culture model and mouse uterine tissue. Slides were dewaxed, target antigen retrieved and permeabilized for 15 min using permeabilization buffer (Dako) at room temperature, followed by blocking in 10% goat serum for 1 h. Primary antibodies against FOXA2 (1:200, ab108422, Abcam, RRID: AB_11157157), Vimentin (1:500, ab20346, Abcam, RRID: AB_445527), ZO‐1 (1:100, 98225S, Cell Signaling), VDR (1:200, ab3508, Abcam, RRID: AB_303857) and WNT7B (1:200, PA5‐103480, Invitrogen, RRID: AB_2852816) were applied overnight at 4 °C, followed by incubation in secondary antibodies (Goat anti‐rabbit IgG (H+L) Alexa Fluor 594 (1:500, A‐11012, Invitrogen, RRID: AB_141359), Goat anti‐rabbit IgG (H+L) Alexa Fluor 488 (1:500, A‐11008, Invitrogen, RRID: AB_143165), Goat anti‐mouse IgG (H+L) Alexa Fluor 488 (1:500, A‐11001, Invitrogen, RRID: AB_2534069) for 1 h in dark. The slides were counterstained with DAPI (1:500, Dako) for 5 min and the fluorescence signal was detected with Carl Zeiss LSM 800 inverted confocal microscope at the Centre for PanorOmic Sciences (CPOS), The University of Hong Kong.

### 2.20 Reverse Transcription Quantitative PCR (RT‐qPCR)

EOs were collected in cell recovery solution, centrifuged at 1000 rcf for 5 min, and resuspended in 900 µl of RNAiso Plus solution for RNA extraction. Reverse transcription was conducted using PrimeScript RT Reagent Kit (TaKaRa). Quantitative PCR (qPCR) was conducted using QuantStudio 5 Real‐Time PCR System (Applied Biosystems) with TaqMan qPCR assay probes (*TEAD4*: Hs01125032_m1; *MMP‐2*: Hs01548727_m1; *HLA‐G*: Hs00365950_g1; *ITGA5*: Hs01547673_m1; *FN1*: Hs01549976_m1; *WNT7B*: Hs00536497_m1, *18S*: Hs99999901_s1, Thermo Fisher Scientific). The reaction was repeated with duplicates, and the threshold cycle (CT) method (2^−△△CT^ method) was used for calculation.

### 2.21 Western Blot Analysis

EOs were collected in RIPA lysis buffer (Thermo Fisher Scientific) with 1% protease inhibitor (CALBIOCHEM) and centrifuged at 12 000 rcf for 30 min at 4 °C for protein extraction. Protein concentration was determined using Pierce BCA protein assay kit. Protein samples were separated on 10% SDS‐polyacrylamide gel by electrophoresis, then transferred to polyvinylidene difluoride membranes. The membrane was blocked with 5% milk for 1 h, then incubated in primary antibody against WNT7B (1:100, AF3460, R&D Systems, RRID: AB_2834898). HRP‐conjugated anti‐goat secondary antibody (1:1000, sc2354, Santa Cruz, RRID: AB_628490) was then applied for 1 h after washing. The membrane was developed with ECL Chemiluminescence reagent (Santa Cruz) using ImageQuant LAS 500 machine (GEHealthcare). The membrane was then stripped using stripping buffer, incubated with primary antibody against β‐actin (1:2000, MA1‐140, Invitrogen, RRID: AB_2536844) overnight at 4 °C followed by Mouse IgG HRP linked whole ab (1:2000, NA931, Cytiva, RRID: AB_772210) for 1 h as internal control.

For the concentrated conditioned medium of the co‐culture model, primary antibodies against PAEP (1:500, ab17247, Abcam), MUC‐1 (1:500, ab109185, Abcam), MMP‐3 (1:500, ab53015, Abcam, RRID: AB_881242) and IL‐6 (1:500, ab9324, Abcam, RRID: AB_307175) were applied, followed by secondary antibodies of mouse IgG HRP‐linked whole antibody (1:2000, NA931, Cytiva, RRID: AB_772210) and rabbit IgG HRP‐linked whole antibody (1:2000, NA934, Cytiva, RRID: AB_772206). After development, the membrane was stained in Coomassie Blue Staining Solution (Thermo Fisher Scientific) for 30 min for protein loading control.

### 2.22 Statistical Analysis

Statistical analysis was conducted using the GraphPad Prism software (Graph pad 9 software Inc.). Data was presented as mean ± standard deviation. All the results were analyzed by the Kolmogrov‐Smirnov normality test. Statistical comparison was conducted using the Student t‐test for two groups or one‐way ANOVA with multiple comparison for more than three groups of variables. *p*<0.05 was considered to be statistically significant.

## 3 Results

### 3.1 Direct Stromal‐Epithelial Interactions in 3D Co‐Culture Promote Endometrial Gland Development and Secretory Function In Vitro

**FIGURE 1:**
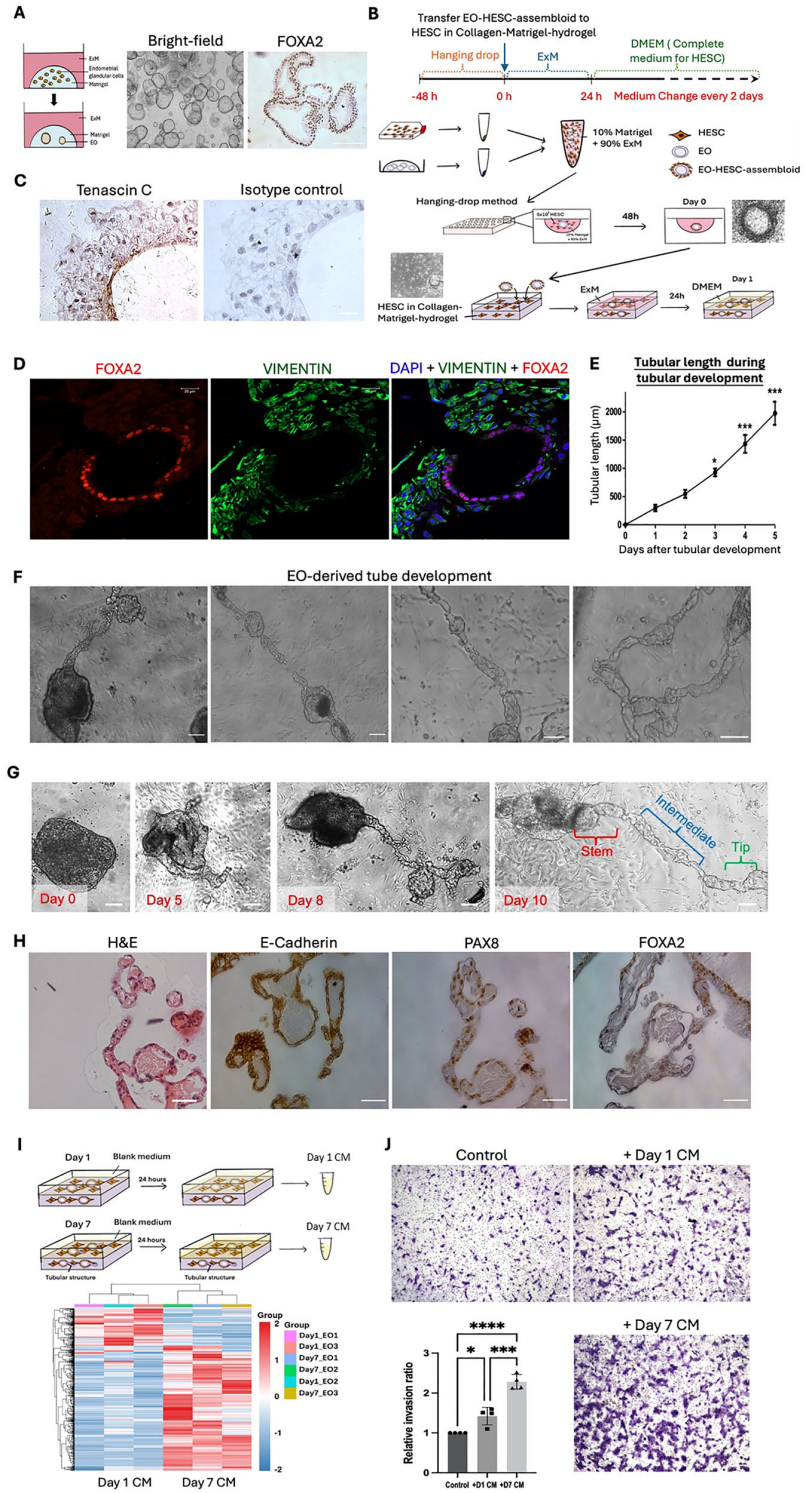
Establishment of 3D endometrial assembloid/human endometrial stromal cell (HESC) co‐culture model with development of assembloid‐derived tubular glands. (A) Schematic illustration for EO model establishment. Bright‐field image of EOs under light microscope. Immunohistochemical staining for FOXA2 in EOs. Scale bars, 100 µm. (B) Schematic illustration and representative bright‐field and H&E staining images for establishment of EO‐HESC‐assembloid and subsequent 3D co‐culture model. ExM: EO expansion medium; DMEM: HESC culture medium. Scale bars, 100 µm. (C) Immunohistochemical staining for Tenascin C in EO‐HESC‐assembloid after hanging‐drop co‐culture. Nuclei were counterstained with Hematoxylin. Scale bars, 25 µm. (D) Immunofluorescence staining for FOXA2 (red) and VIMENTIN (green) in day‐2 EO/HESC co‐culture model. Nuclei were counterstained with DAPI (blue). Scale bars, 20 µm. (E) Average length of assembloid‐derived tubular gland during tubular development compared to day 1. *: *p*<0.05, ***: *p*<0.001. *n* = 4. (F) Representative bright‐field images for assembloid‐derived tubular gland. Scale bars, 100 µm. (G) Representative bright‐field images for assembloid‐derived tubular glands on day 0, day 5, day 8 and day 10 after culture. Scale bars, 50 µm. (H) H&E and Immunohistochemical staining for E‐Cadherin, PAX8 and FOXA2 in assembloid‐derived tubular gland. Nuclei were counterstained with Haematoxylin. Scale bars, 100 µm. (I) Schematic illustration for conditioned medium (CM) collection from 3D EO/HESC co‐culture model before (day 1) and after (day 7) assembloid‐derived tubular gland development. Clustered heatmap for differentially expressed proteins (DEPs) from conditioned medium collected from day 1 3D model (Day 1 CM) and day 7 3D model (Day 7 CM). DEPs were generated from Day 7 CM versus Day 1 CM. *n* = 3. (J) Invasion assay for EVTs treated with 30 µg of Day 1/Day 7 CM for 20 h. Representative images for invaded EVTs treated with vehicle control (control), Day 1 CM (+Day1 CM) and Day 7 CM (+Day7 CM) with relative invasion ratio. *: *p*<0.05, ***: *p*<0.001, ****: *p*<0.0001. Scale bars, 100 µm. *n* = 4. All data was presented as mean ± standard deviation. All the results were analyzed by the Kolmogrov‐Smirnov normality test. Statistical comparison was conducted using the Student t‐test for two groups or one‐way ANOVA with multiple comparison for more than three groups of variables.

To study the development of endometrial glands in vitro, we established Forkhead Box A2 (FOXA2)‐positive EOs to mimic the morphology and molecular characteristics of human endometrial glands (Figure 1A). These organoids consisted of a sphere‐like cell layer enclosing a lumen consistent with previous reports [15, 33], which were capable of long‐term growth and expansion, and recapitulated the molecular features of the endometrial glands by expressing key glandular and epithelial markers, including *FOXA2*, E‐cadherin, Progestagen associated endometrial protein (*PAEP*), Paired Box 8 (*PAX8*), and SRY‐Box Transcription Factor 17 (*SOX17*) [33]. The EOs were co‐cultured with HESCs in hanging‐drops for 48 h to allow direct epithelial cell‐HESC contact. The resultant EO‐HESC‐assembloids were embedded in HESC‐suspended collagen‐Matrigel‐hydrogel scaffold (Figure 1B).

The physical and chemical communication between cell types forms the foundation for tissue development and function. Interactions between the stromal cells and the epithelial cells can occur via ECM. For example, the ECM molecule Tenascin C is exclusively expressed in mesenchymal‐surrounded epithelial cells, which influences morphological changes of epithelium by interacting with ECM receptors [23, 53, 54]. In this model, the EOs in the assembloids expressed strong immunoreactivity for Tenascin C, indicating robust mesenchymal‐epithelial interactions (Figure 1C). Meanwhile, expression of tight junction marker ZO‐1 has been found in the EO/HESC assembloid, indicating the cell‐cell interaction during the hanging‐drop coculture (Figure S1A). After 2 days of co‐culture, the EOs in the assembloids maintained expression of the endometrial gland marker FOXA2, while the HESCs expressed the mesenchymal marker VIMENTIN (Figure 1D).

Most importantly, the EOs in the assembloids developed tubular structures by Day 6‐7, recapitulating the human endometrial glands morphologically. The tubular structures lengthened continuously during the following five‐days of culture (Figure 1E–G; Figure S1B). The assembloid‐derived tubular gland expressed the epithelial marker E‐Cadherin and the endometrial gland markers PAX8 and FOXA2, confirming their glandular origin (Figure 1H). In this study, we derived EOs from 8 human endometrial samples. Interestingly, only EOs from 4 samples displayed tube‐formation capacity. There were no obvious demographic differences between the donors of samples with and without tube‐formation capacity (Table S1).

To investigate whether EO‐HESC interactions in the hanging‐drops induced tube formation of the assembloids, EOs were cultured alone without HESCs (EO only) or co‐cultured with HESCs in collagen‐Matrigel‐hydrogel scaffold without prior assembloid formation in the hanging‐drops (simple co‐culture; Figure S1C). The EOs in the assembloids showed significantly higher viability than those in EOs alone culture and those in simple co‐culture with HESCs, in terms of the compaction of morphology (Figure S1D). Neither the culture of EO alone nor simple HESC co‐culture induced tube formation (Figure S1E). These observations highlight the importance of direct HESCs contact in the viability and tube formation of EOs.

To examine the secretory function of the assembloid‐derived tubular gland, proteomic analyses of the conditioned media collected from the co‐culture model before (day 1) and after tube formation (day 7) were conducted and differentially expressed proteins (DEPs) were identified (Figure 1I; supporting data). Gene Ontology (GO) analysis revealed the functions of the DEPs in extracellular matrix/structure organization, protease activities and immune modulation (Figure S2A). Four DEPs, including PAEP, Mucin‐1 (MUC‐1), Matrix metalloproteinase‐3 (MMP‐3) and Interleukin‐6 (IL‐6), were shortlisted because of their high expression in the proteomics analyses and known function as endometrial glandular secretory products, which expression levels did not vary in conditioned medium collected from HESC‐only culture on day 7 and day 1 (Figure S2B). Western blotting confirmed their elevated secretion in the Day 7 conditioned medium from co‐culture model (Figure S2C). All the 4 DEPs showed strong and exclusive expression in the endometrial glands of human endometrial tissue in the secretory phase (Figure S2D). Functionally, trophoblast invasion assays demonstrated that the Day 7 conditioned medium enhanced the invasiveness of human trophoblast stem cells (hTSC, Figure S2E)‐derived extravillous trophoblasts (EVTs) compared with the Day 1 conditioned medium (Figure 1J), consistent with the regulatory role of endometrial glands on trophoblast functions. Overall, an endometrial assembloid model capable of developing tubular structures mimicking endometrial gland development was established. The model demonstrates a role of direct stromal‐epithelial interactions in the development and function of endometrial glands in vitro.

### 3.2 EOs Demonstrate Unique Gene Expression Profiles during Tube Formation

**FIGURE 2:**
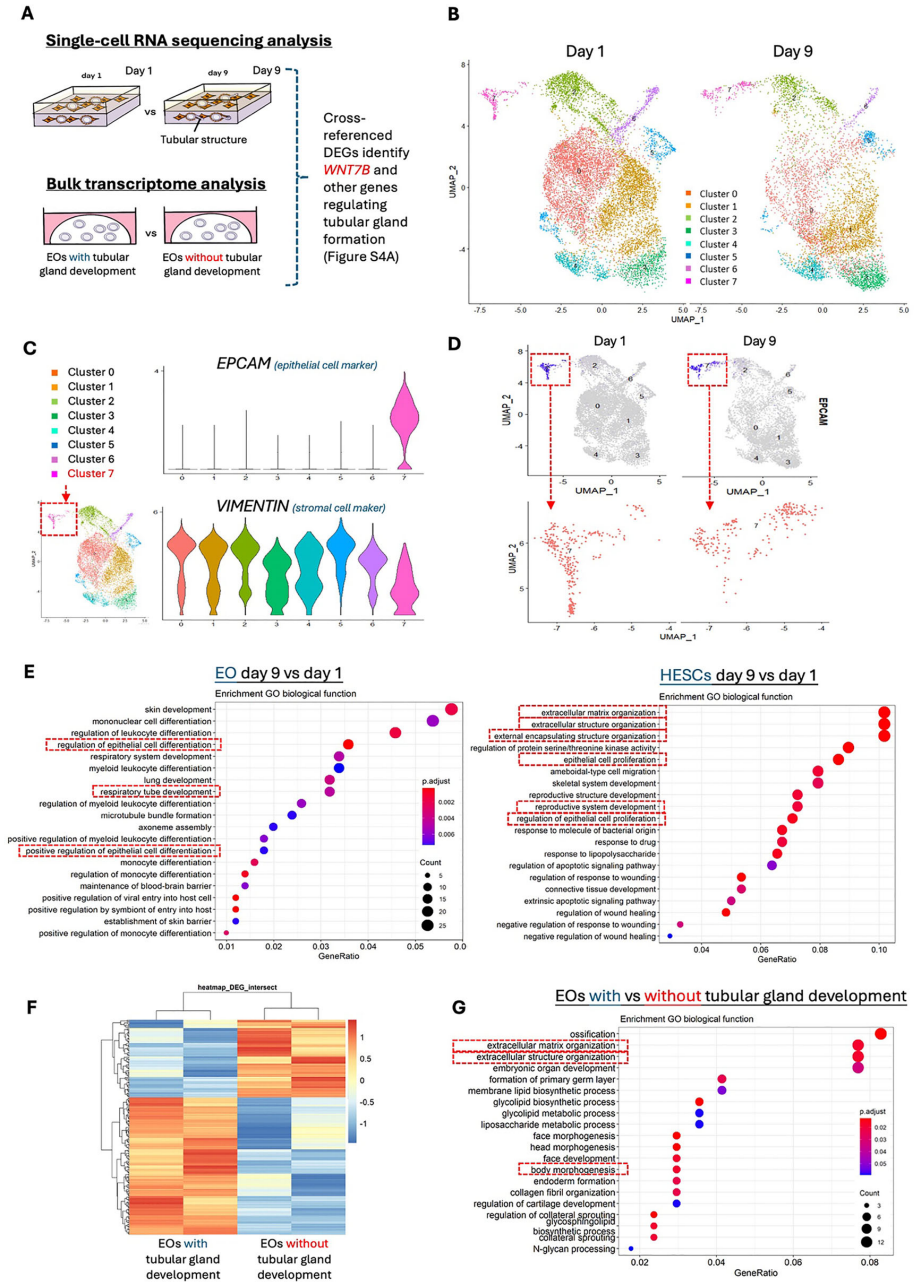
Transcriptome analyses for tubular gland development from EOs. (A) Schematic illustration for single‐cell RNA sequencing analysis and bulk transcriptome analysis for identifying WNT7B and other genes regulating tubular gland formation (Figure S4A). (B) UMAP plot for the 3D EO/HESC co‐culture model on day 1 and day 9 in single‐cell RNA sequencing analysis. Colors indicate different cellular clusters (0–7). (C) Violin plot for *EPCAM* (epithelial marker) and *VIMENTIN* (fibroblast marker) expression in cellular clusters 0–7. (D) UMAP plot for *EPCAM* expression in day 1 and day 9 EO/HESC co‐culture model and zoomed UMAP plot for Cluster 7 on day 1 and day 9. (E) Enriched GO analysis of biological functions for differentially expressed genes (DEGs) identified in EOs (Cluster 7) and HESCs (Cluster 0–6) on day 9 versus day 1. The red boxes highlight the biological functions related to gland development. (F) Clustered heatmap for DEGs in EOs with and without tubular gland development capacity in bulk transcriptome analysis. *n* = 2. (G) Enriched GO analysis of biological functions for DEGs in EOs with and without tubular gland development capacity. The red boxes highlight the biological functions related to gland development.

To investigate the factors influencing tube formation in EOs, we conducted two sequencing analyses to identify the genetic regulation involved in tubular gland development. These analyses included a single‐cell RNA sequencing analysis comparing genetic signatures before (day 1) and after tube development (day 9), and a bulk transcriptome analysis comparing EOs with and without tube‐formation capacity (Figure 2A).

The single‐cell RNA sequencing on the co‐cultured cells before and after tube formation identified eight cell clusters (Figure 2B). Clusters 0‐6 contained the co‐cultured HESCs with high *VIMENTIN* expression, while Cluster 7 contained the EO/tubular cells with strong *EPCAM* expression (Figure 2C). Notably, Cluster 7 exhibited a differential cell distribution before and after tube formation, consistent with the dynamic morphological transition during tube development (Figure 2D). Gene Ontology (GO) analyses for the EO and HESC clusters identified differentially expressed genes (DEGs) relevant to gland development, respectively. These covered, for example, the regulation of epithelial differentiation in EOs, and ECM and extracellular structure organization in HESCs (Figure 2E).

Mapping these clusters to a published single‐cell endometrial dataset for human full‐thickness endometrial tissue [48] confirmed that the assembloid–derived tubular gland cells (Cluster 7) corresponded to the glandular epithelium in vivo. We extracted the endometrial gland cells (Glandular Epithelia) and endometrial stromal cells (eS) from the published dataset. By regressing out the batch effect and integrating the data, we observed that the Cluster 7 from Day 1 and Day 9 matched with the Glandular Epithelia cluster from human endometrium under UMAP projection. In contrast, other cell clusters aligned with the eS region (Figure S3). These findings validate the physiological relevance of our in vitro co‐culture model to the human endometrium and reveal distinct gene expression profiles of the EOs after tube formation.

The bulk RNA sequencing revealed a distinct transcriptomic signature in EOs with and without tube‐formation capacity (Figure 2F). GO analysis of DEGs indicated their involvement in biological processes related to ECM organization, extracellular structure organization and morphogenesis (Figure 2G).

### 3.3 Downregulation of *WNT7B* Promotes Tubular Gland Formation of EOs

**FIGURE 3:**
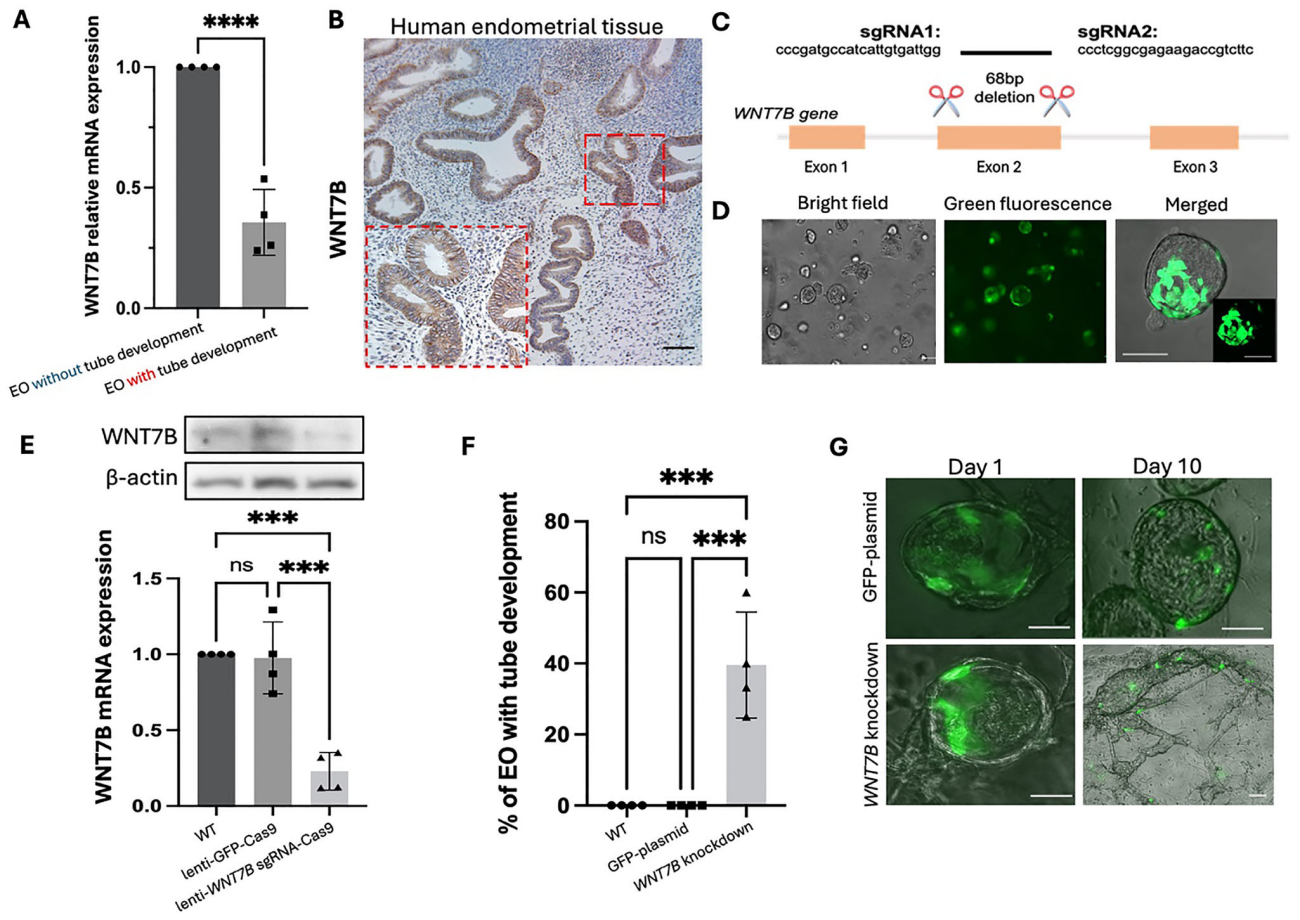
Reduced *WNT7B* expression stimulates assembloid‐derived tubular gland development. (A) RT‐qPCR analysis for relative mRNA expression level of *WNT7B* in EOs with/without tubular gland development capacity. ****: *p*<0.0001. *n* = 4. (B) Immunohistochemical staining for WNT7B in human endometrial tissue. Nuclei were counterstained with Hematoxylin. Red box: magnified section of the field. Scale bars, 100 µm. (C) Schematic illustration for CRISPR Cas9 system targeting *WNT7B* exon 2. (D) Representative images of EOs after *WNT7B*‐targeted lentivirus transduction for CRISPR Cas9. Green fluorescence indicates GFP expression. Scale bars, 100 µm. (E) Western blot analysis (upper) and RT‐qPCR analysis (lower) for *WNT7B* protein and mRNA expression levels in wildtype EOs (WT), non‐targeting lentivirus transduced EOs (lenti‐GFP‐Cas9) and *WNT7B* knockdown EOs (lenti‐*WNT7B* sgRNA‐Cas9). ***: *p*<0.001. ns: not significant. *n* = 4. (F) Proportion of *WNT7B* knockdown EOs with tubular gland formation compared to wildtype EOs (WT) and non‐targeting lentivirus transduced EOs (GFP‐plasmid). ***: *p*<0.001. ns: not significant. *n* = 4. (G) Representative images of non‐targeting lentivirus transduced EOs (GFP‐plasmid) and *WNT7B* knockdown EOs in the 3D co‐culture model on day 1 and day 10. Merged images were generated from bright‐field image plus green fluorescence image. Scale bars, 100 µm. All data was presented as mean ± standard deviation. All the results were analyzed by the Kolmogrov‐Smirnov normality test. Statistical comparison was conducted using the Student t‐test for two groups or one‐way ANOVA with multiple comparison for more than three groups of variables.

To identify the genes that regulate tube formation of EOs, we cross‐referenced the DEGs from bulk sequencing and single‐cell RNA sequencing analyses, focusing on those with the same expression pattern in both analyses (Figure S4A). Of these DEGs, we confirmed the downregulation of *WNT7B*, *KCTD12*, and *RGS22* in EOs related to tube formation by RT‐qPCR (Figure 3A; Figure S4B).

Immunohistochemical staining revealed strong WNT7B expression in the endometrial glands of human endometrial biopsies (Figure 3B). We hypothesized that a reduction of *WNT7B* expression induced tube formation in EOs. To test the hypothesis, a CRISPR Cas9‐based knockdown strategy targeting exon 2 of *WNT7B* was performed on EOs previously incapable of tube formation (Figure 3C). Using our established protocol [33, 35] we successfully transduced EOs with GFP‐tagged lentivirus (Figure 3D). The EOs treated with *WNT7B*‐targeting sgRNA lentivirus exhibited substantial reduction in WNT7B mRNA and protein levels compared to the wild‐type EOs and those treated with non‐targeting sgRNA lentivirus (Figure 3E). After assembloid formation and co‐culture with HESCs, *WNT7B* knockdown EOs showed a significantly higher rate of tube formation than the two controls, which displayed no morphological changes (Figure 3F,G). The findings indicate that the downregulation of *WNT7B* is crucial for tube formation in EO‐HESC‐assembloids.

### 3.4 HESC‐Derived TGFβ1 Regulates *WNT7B* Expression and Tube Formation of EOs

**FIGURE 4:**
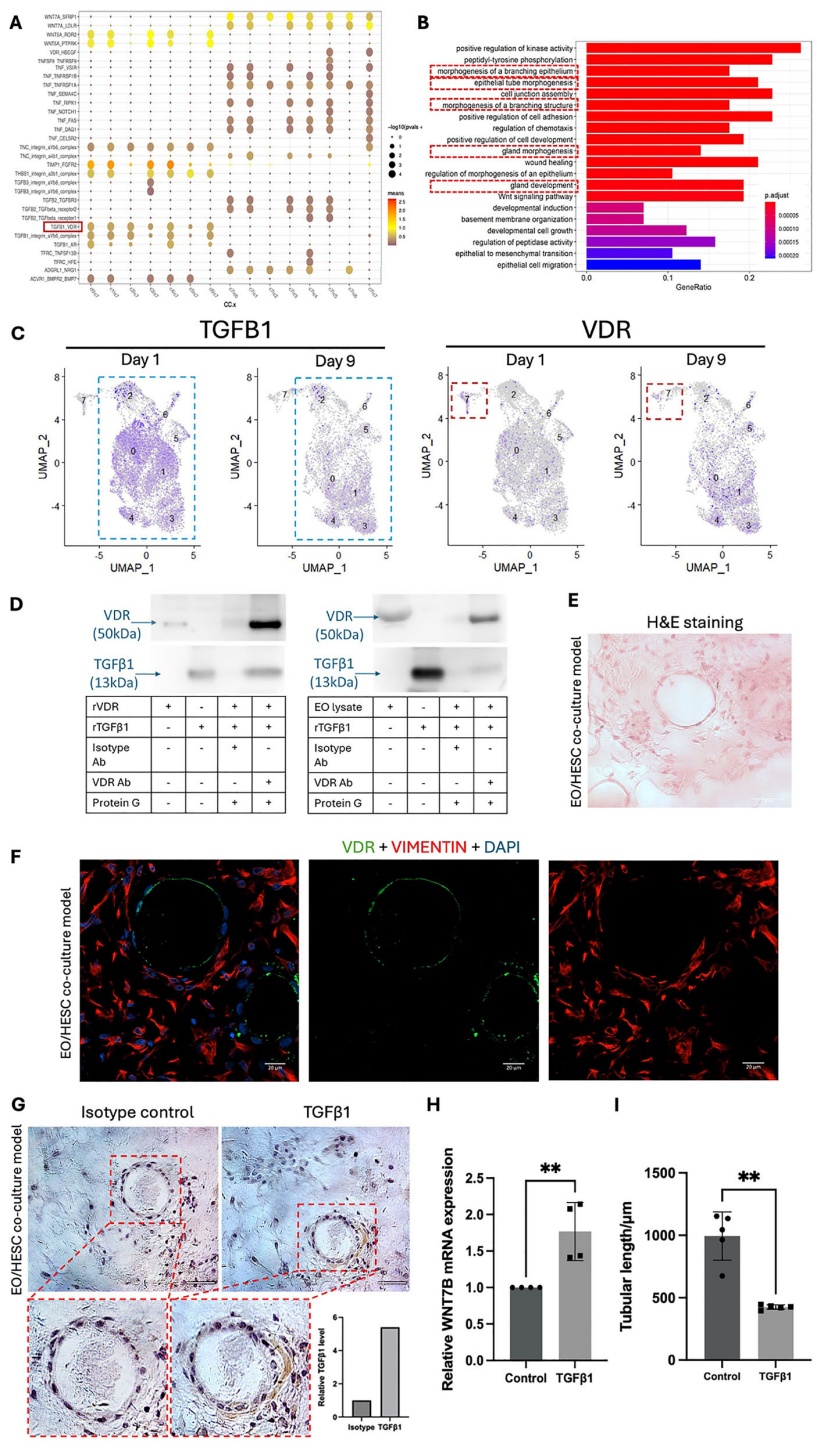
TGFβ1‐VDR interaction between HESCs and EOs regulates *WNT7B* expression and assembloid‐derived tubular gland development. (A) Dot plot for predicted ligand‐receptor pairs between HESCs (Cluster 0–6) and EOs (Cluster 7) according to single‐cell RNA sequencing analysis. The red box highlights TGFβ1‐VDR interaction. (B) Enriched GO analysis of biological functions in ligand‐receptor pairs between EOs and HESCs by CellPhone DB. The red boxes highlight the terms related to gland development, glandular structure morphogenesis and epithelium branching. (C) UMAP plot for TGFβ1 and VDR expression in single‐cell RNA sequencing analysis of day 1 and day 9 3D model. The red box highlights Cluster 7 (EOs/tubular EOs); blue box highlights Cluster 0‐6 (stromal cells). (D) Recombinant VDR or lysate of EO were extracted and were incubated with recombinant TGFβ1. Anti‐VDR and Protein G beads were used to co‐immunoprecipitated the VDR‐interacting protein complexes. The captured complex was analyzed by Western blotting using anti‐TGFβ1 and ‐VDR antibodies. *n* = 3. Figure shows a representative image from three independent experiments. (E) H&E staining of 3D EO/HESC co‐culture model on day 1 after co‐culture. Scale bars, 100 µm (top) and 50 µm (bottom). (F) Immunofluorescence staining for VDR (green) and VIMENTIN (red, fibroblast marker) in 3D EO/HESC co‐culture model on day 1 after co‐culture. Nuclei were counterstained with DAPI (blue). Scale bars, 20 µm. (G) Immunohistochemical staining for TGFβ1 in 3D EO/HESC co‐culture model on day 1 after co‐culture. Nuclei were counterstained with Hematoxylin. The bar chart indicates the quantified staining intensity of TGFβ1 in comparison to isotype control. Scale bars, 50 µm. (H) RT‐qPCR analysis for relative mRNA expression level of *WNT7B* in EOs after TGFβ1 treatment at 10 ng mL^−1^ for 48 h. **: *p*<0.01. *n* = 4. (I) Average tubular length of assembloid‐derived tubular structure in the co‐culture model from EOs pretreated with 10 ng mL^−1^ of TGFβ1 for 48 h. **: *p*<0.01. *n* = 5. All data was presented as mean ± standard deviation. All the results were analyzed by the Kolmogrov‐Smirnov normality test. Statistical comparison was conducted using the Student t‐test for two groups or one‐way ANOVA with multiple comparison for more than three groups of variables.

Next, we investigated the extrinsic regulation of tube formation by HESCs. Ligand‐receptor analysis using CellPhone DB identified 280 potential ligand‐receptor pairs between co‐cultured EOs and HESCs (Figure 4A). GO analysis revealed that many of these pairs are involved in gland development, glandular structure morphogenesis and epithelial branching (Figure 4B). Among them, the interaction between Vitamin D receptor (*VDR*) in EOs and *TGFβ1* in HESCs was selected for further study due to its known link to Wnt signaling pathway [55, 56].

Our single‐cell RNA sequencing data showed reduced expression levels of both *VDR* and *TGFβ1* after tube formation in EO/tubular cells and HESC clusters, respectively (Figure 4C). The reduction levels of both VDR and TGFβ1 also coincided with decreased *WNT7B* expression in EOs after tube formation. The interaction between VDR and TGFβ1 was also demonstrated by co‐immunoprecipitation using recombinant protein and EO cell lysate (Figure 4D; Figure S5A). To validate these expression patterns in vitro, we collected EOs and HESCs after one day of co‐culture (Figure 4E) and confirmed positive VDR expression in EOs (Figure 4F) and both expression and secretion of TGFβ1 in HESCs (Figure 4G; Figure S5B). Treatment of EOs with exogenous TGFβ1 (10 ng mL^−1^) for 48 h significantly increased *WNT7B* mRNA expression compared to vehicle control (Figure 4H). Coincidentally, the tube length was reduced in the co‐culture model compared to vehicle control after 10 days (Figure 4I). Moreover, inhibition of VDR via the action of VDR blocker TEI‐9648 significantly reduced the expression of TGFβ1‐induced WNT7B expression, further demonstrating the TGFβ1‐VDR signaling in WNT7B expression (Figure S5C). These data suggest that stromal TGFβ1‐epithelial VDR signaling regulates *WNT7B* levels in EOs, and thereby modulates tube formation.

### 3.5 Endometrial‐Specific WNT7B Ablation Enhances Mouse Endometrial Gland Development

**FIGURE 5:**
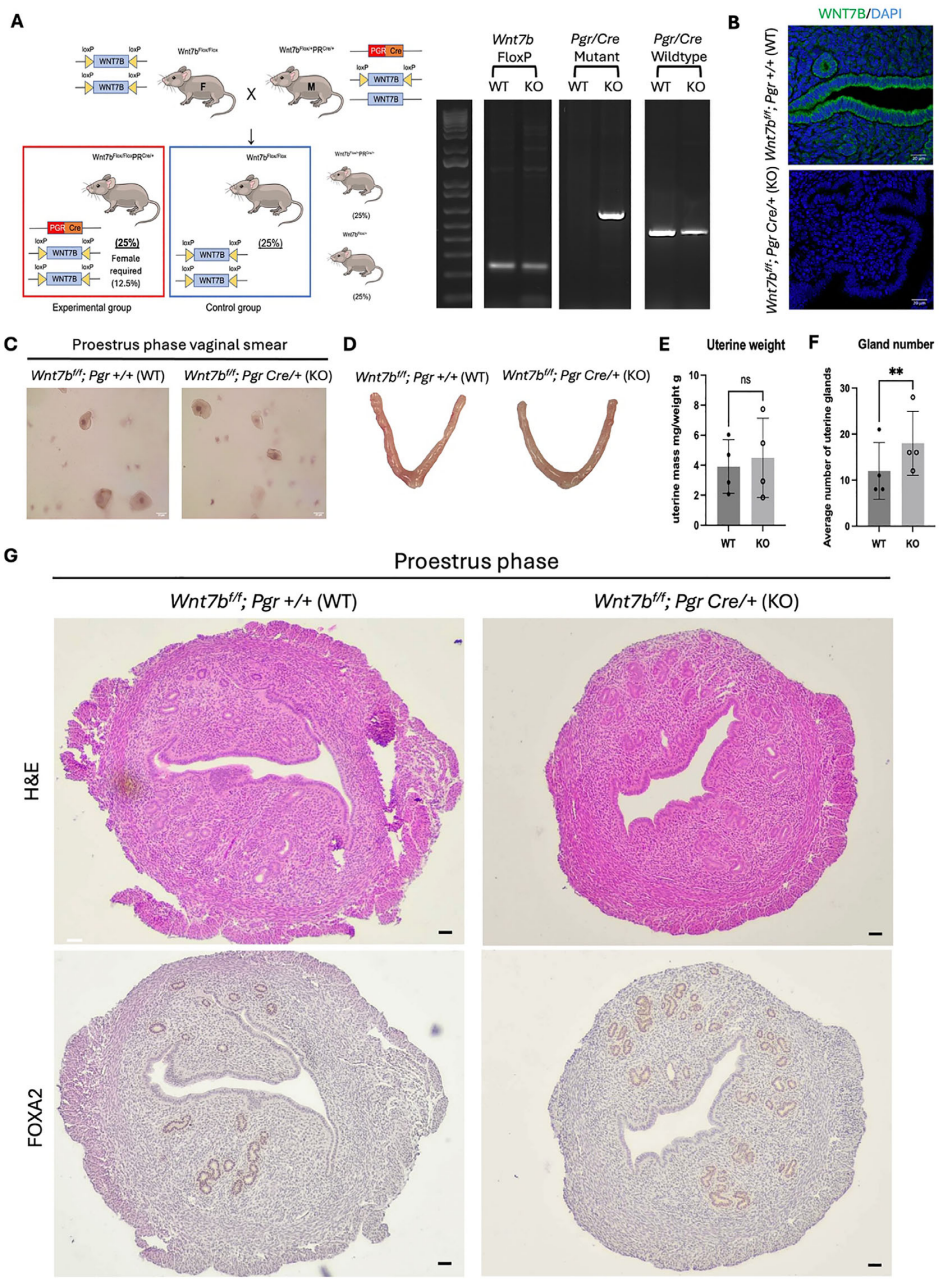
Ablation of *WNT7B* expression in mouse endometrium results in increased endometrial gland number at proestrus phase. (A) Schematic illustration for establishment of endometrial‐specific WNT7B knockout mouse by Cre‐loxP system. Genotyping for *Wnt7b^f/f^; Pgr Cre/+* mouse via PCR and gel electrophoresis. WT: wildtype control (*Wnt7b^f/f^; Pgr +/+*); KO: conditional knockout (*Wnt7b^f/f^; Pgr Cre/+*). (B) Expression of WNT7B (green) in mouse endometrium in wildtype and conditional knockout mice. Nuclei were counterstained with DAPI (blue). Scale bars, 20 µm. (C) Vaginal smear staining for mouse at proestrus phase. Nuclei were counterstained with Hematoxylin. Scale bars, 25 µm. (D) Representative images for uteri of wildtype mouse (WT) and *WNT7B* conditional knockout mouse (KO) at 6 weeks at proestrus phase. (E) Uterine weight for wildtype (WT) and *WNT7B* conditional knockout (KO) mice at 6 weeks at proestrus phase. The uterine weight was divided by the body weight. ns: not significant. *n* = 4. (F) Average endometrial gland number in wildtype (WT) and *WNT7B* conditional knockout (KO) mice at 6 weeks at proestrus phase. **: *p*<0.01. *n* = 4. (G) H&E staining and immunohistochemical staining for FOXA2 in endometrial tissue of wildtype (WT) and *WNT7B* conditional knockout (KO) mice at 6 weeks at proestrus phase. Nuclei were counterstained with Hematoxylin. Scale bars, 100 µm. All data was presented as mean ± standard deviation. All the results were analyzed by the Kolmogrov‐Smirnov normality test. Statistical comparison was conducted using the Student t‐test for two groups or one‐way ANOVA with multiple comparison for more than three groups of variables.

To functionally validate the role of *WNT7B* in endometrial gland development, we produced endometrial‐specific *WNT7B* knockout mice (*Wnt7bf/f; Pgr Cre/+*) using the Cre‐loxP system. This was achieved by crossing mice expressing Cre recombinase under the progesterone receptor promoter (*Pgrtm1.1(cre)Shah*) with mice containing a floxed *Wnt7b* allele (*Wnt7btm2Amc*) (Figure 5A). Consequently, cells expressing the progesterone receptor exhibited ablated *WNT7B* expression, resulting in an endometrium‐specific *WNT7B* knockout genotype. Immunofluorescence staining confirmed the absence of WNT7B expression in the luminal and glandular epithelium of knockout mice (Figure 5B).

We sacrificed the endometrial‐specific *WNT7B* knockout mice (*Wnt7bf/f; Pgr Cre/+*) and wild‐type mice (*Wnt7bf/f; Pgr +/+*) at 6 weeks of age in their proestrus stage as determined by vaginal smears (Figure 5C). There were no significant differences in uterine size or uterine‐to‐body weight ratio between the wild‐type and the knockout mice (Figure 5D,E). However, the knockout mice exhibited a significantly higher number of FOXA2‐positive endometrial glands compared to the wild‐type group (Figure 5F,G). While some variation in gland counts between serial sections may occur due to technical challenges in histological processing and the complex 3D architecture of endometrial glands, our quantitative analysis was based on comprehensive assessment across multiple sections from each animal. In conclusion, the endometrial‐specific ablation of *WNT7B* expression leads to exaggerated endometrial gland development in the proestrus phase, when serum estradiol concentration is high. The finding suggests the role of *WNT7B* as an inhibitor in endometrial gland development.

### 3.6 Estradiol Enhances Tube Formation of EOs by Downregulation of *WNT7B*

**FIGURE 6:**
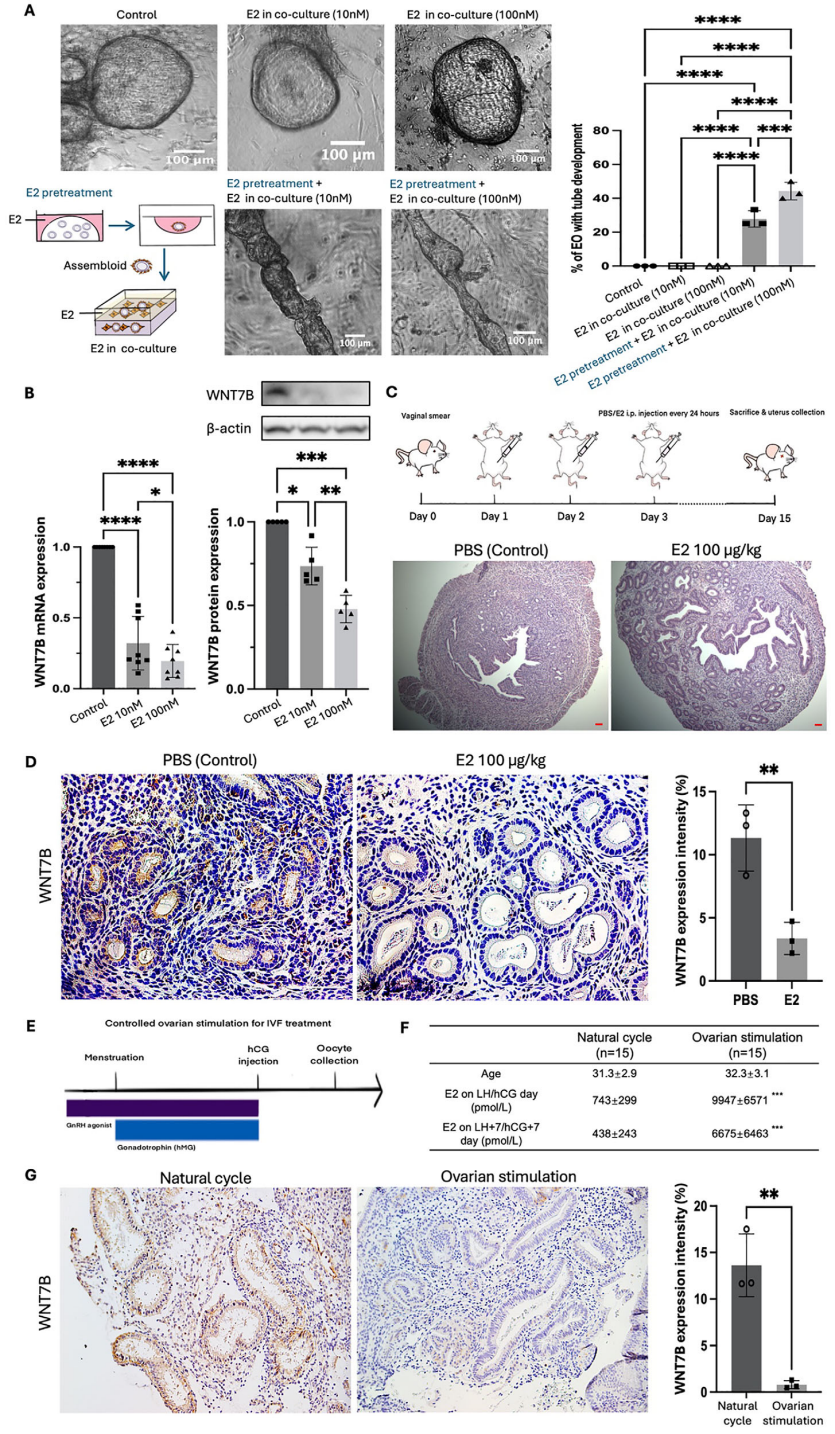
Estradiol stimulates endometrial gland development with *WNT7B* downregulation in assembloid‐derived tubular gland, estradiol stimulated mouse and human IVF clinical models. (A) Proportion of EOs with tubular gland formation in estradiol (E2) pretreatment and E2 treatment in HESC/assembloid co‐culture model. Scale bars, 100 µm. ***: *p*<0.001; ****: *p*<0.0001. *n* = 3. (B) Western blot analysis (*n* = 5, left) and RT‐qPCR analysis (*n* = 8, right) and for relative protein and mRNA expression level of *WNT7B* in wildtype EOs (control) and EOs treated with 10 nM/100 nM of estradiol (E2) for 7 days. *: *p*<0.05; **: *p*<0.01; ***: *p*<0.001****: *p*<0.0001. *n* = 8. (C) Schematic illustration for estradiol‐stimulated mouse model establishment. H&E staining for endometrial sections of mice with i.p. PBS injection (control) or 100 µg kg^−1^ of estradiol injection (E2) for 14 days. i.p.: Intraperitoneal. PBS: PBS injection for control group; E2: estradiol injection. Scale bars, 100 µm. Dosing timeline: Estrous‐cycle monitoring for approximately 2 weeks prior to day 0 is not depicted. Day 0: proestrus confirmed by late‐afternoon vaginal smear; day 1–14: daily i.p. PBS or 100 µg kg^−1^ E2; day 15: tissue collection. (D) Immunohistochemical staining for WNT7B in endometrial sections of mice with i.p. PBS injection (control) or 100 µg kg^−1^ of estradiol injection (E2) with quantified expression intensity. Nuclei were counterstained with Hematoxylin. **: *p*<0.01. Scale bars, 100 µm. *n* = 3. (E) Schematic illustration for controlled ovarian stimulation protocol in female participants undergoing IVF treatment. GnRH: gonadotrophin releasing hormone; hMG: human menopausal gonadotrophin. (F) Age and serum estradiol concentrations in female participants with natural menstrual cycle and controlled ovarian stimulation for IVF treatment. Data represents mean ± standard deviation. ***: *p*<0.001. *n* = 15. (G) Immunohistochemical staining for WNT7B in endometrial tissue of female participants with natural menstrual cycle and controlled ovarian stimulation with quantified expression intensity. Nuclei were counterstained with Hematoxylin. **: *p*<0.01. Scale bars, 100 µm. *n* = 3. All data was presented as mean ± standard deviation. All the results were analyzed by the Kolmogrov‐Smirnov normality test. Statistical comparison was conducted using the Student t‐test for two groups or one‐way ANOVA with multiple comparison for more than three groups of variables.

It is well‐known that estradiol induces endometrial gland development in the proliferative phase of menstrual cycles [57]. Therefore, EOs were treated with 10 or 100 nM estradiol for 7 days. The treated EOs exhibited a dose‐dependent increase in estrogen receptor 1 (ESR1) expression, confirming their hormone responsiveness (Figure S6A,B). After 7 days of estradiol treatment at 10 or 100 nM followed by co‐culture with HESCs in our 3D model, EOs that were previously unable to form tubes acquired tube‐forming capacity (Figure 6A). In contrast, EOs without estradiol treatment or those without estradiol treatment during co‐culture but not in assembloid formation did not develop tubular structures. Estradiol treatment at 10 or 100 nM during assembloid formation resulted in reduced WNT7B expression at both the transcript and protein levels (Figure 6B), which correlated with the degree of tube formation. These findings suggest that estradiol promotes EO tube formation in vitro by downregulation of *WNT7B* expression.

### 3.7 High Estradiol Reduces *WNT7B* Expression and Promotes Endometrial Gland Development In Vivo

To validate the in vitro findings in an in vivo context, a model of sustained estradiol stimulation in intact cycling mice was developed. In this model, female mice at proestrus phase as determined by vaginal smear were used (Figure S7A). They received intraperitoneal injections of 100 µg kg^−1^ of estradiol every 24 h for 14 days. The physiological baseline control group was injected with the same volume of phosphate buffered saline (PBS) (Figure 6C). After 14 days, the uteri of the estradiol‐treated mice were larger in size and significantly heavier than the control group (Figure S7B,C). Haematoxylin and eosin staining showed that the estradiol‐treated mice had significantly higher number and larger size of endometrial glands than the control mice (Figure 6C; Figure S7D). Immunohistochemical staining revealed reduced WNT7B expression in the endometrial glands of the estradiol‐treated mice, consistent with the in vitro findings (Figure 6D).

To conduct a complementary human study, we compared endometria from women undergoing controlled ovarian stimulation for IVF and those in natural cycles (Figure 6E). The ages of the women in both groups were comparable. Women in stimulated cycles had significantly higher serum estradiol concentrations on the day of hCG injection and seven days later, compared to women in natural cycles at the corresponding time points (LH surge day and seven days after LH surge; Figure 6F). Immunohistochemical staining of endometrial biopsies revealed significantly lower WNT7B expression in the endometrial glands of women undergoing controlled ovarian stimulation compared to those in natural cycles (Figure 6G). To further relate to our finding that TGFβ1 regulates WNT7B expression, endometrial biopsies from women undergoing controlled ovarian stimulation presented a significantly lower TGFβ1 expression in their stromal cells (Figure S8A,B). The above findings further suggest that high estradiol levels are associated with reduced WNT7B expression in endometrial glands, which is likely through TGFβ1 regulation.

## 4 Discussion

Endometrial glands are essential for reproductive function and undergo monthly remodeling during each menstrual cycle. This process involves estradiol‐driven elongation, branching, and regeneration of the glands, as well as dynamic crosstalk between stromal and epithelial cells [4, 5]. Yet the mechanisms underlying their formation, development and regeneration remain poorly understood. The development of an effective model to study this process represents an important advancement in reproductive biology research. In this study, the endometrial assembloid model incorporating human EOs and HESCs successfully replicates the formation of tubular gland‐like structures in vitro. Unlike previous studies that construct endometrial assembloids by directly co‐culture of EOs and stromal cells in hydrogel [58], this study employed an initial hanging‐drop culture step to ensure direct physiological interaction between the EOs and HESCs before further contact with the hydrogel matrix. Using these assembloids alongside in vivo and clinical studies, reduced expression of *WNT7B* was identified as an intrinsic promoting factor of tubular glands formation. This process is extrinsically regulated by estradiol and TGFβ1‐VDR signaling between EOs and HESCs.

Extensive studies have shown the impact of endometrial gland secretion on decidualization of HESCs during the menstrual cycle and early pregnancy [2, 59, 60, 61, 62]. However, the relationship between stromal cells and gland development remains poorly understood. Gland development involves protrusion, elongation, and branching of epithelial buds [63]. Organoids derived from several epithelial tissues have demonstrated gland development in 3D culture systems. For instance, murine mammary epithelial cell‐derived organoids exhibit in vitro adenogenesis when cultured in collagen and/or Matrigel in the presence of growth factors [64, 65]. Similarly, organoids from murine pancreatic ductal adenocarcinoma cells show ductal development in a collagen gel [66]. Prostate gland organoids also develop branching phenotypes after direct cell‐cell interactions with prostate stromal cells in a collagen‐Matrigel‐hydrogel [67]. In the assembloid model described here, tubular gland‐like structures were triggered by direct interaction between the epithelial cells and the stromal cells in hanging‐drops. Such interaction is essential for the development of tubular endometrial epithelial glands [68] and other glands in vivo [7, 69], highlighting the importance of epithelial‐stromal crosstalk in human endometrial gland development. While our assembloids complement recent endometrial co‐culture models that replicate hormonal responses, secretory functions and implantation, our key innovation lies in developing the first assembloid system that spontaneously self‐organizes lumenized tubular glands within a 3D stromal matrix, recapitulating the structural hierarchy of the human endometrial gland. This model provides new insights into the structural determinants of endometrial gland that were previously inaccessible. Thus, beyond hormonal receptivity or secretion, our assembloid provides a morphologically defined platform to dissect structural determinants of endometrial pathology such as implantation failure and miscarriage.

Despite using standardized protocols, biological variation among patients introduces important considerations for interpreting research outcomes. In this study, the tube‐forming ability of EOs was patient‐specific, with half of the samples demonstrating this capability in the assembloids. Since the recruited subjects underwent IVF treatment, it was essential to exclude the possibility that these observations resulted from underlying endometrial pathology. Demographic data revealed that the most participants underwent IVF due to male factor infertility, with only one participant having unexplained infertility. None of the donors exhibited endometrial pathology, and the general characteristics of the women did not differ between donors whose EOs could or could not from tubular structure. This variability presented a valuable opportunity to investigate the molecular basis of tube formation. To identify the genetic factors governing these differences, a two‐pronged approach was implemented: first, bulk transcriptome analysis compared EOs with and without tube‐formation capacity; second, single‐cell RNA sequencing examined genetic signatures before and after tube development.

Cross‐referencing these datasets indicate that reduced expression of *WNT7B* is an intrinsic regulatory factor of tube formation in EO. *WNT7B* regulates cell proliferation, differentiation, and migration, and is expressed in endometrial epithelial cells in mice and humans [70]. However, its role in endometrial gland development has not been reported. In the endometrium, several Wnt factors, including Wnt4, Wnt5a, and Wnt7a are critical for endometrial epithelial patterning and gland development [71, 72, 73]. Wnt factors also stimulate tube formation in intestinal gland organoids [26]. High *WNT7B* expression is observed in the terminal bud epithelium during mammary gland development, suggesting its regulatory role in adenogenesis [74]. Genetic manipulation provides definitive evidence for the functional significance of WNT7B in endometrial gland formation. *WNT7B* knockout mouse line specifically in the mouse uteri resulted in an increased number of endometrial glands during the proestrus stage, when the serum and local estradiol levels were highest [75, 76]. During mouse endometrial gland development, differential expression of Wnt factors correlates with asymmetric glandular structure formation [21]. Another study found that endometrial‐specific knockout of *WNT7A* leads to a lack of endometrial glands and reduced fertility in mice [77]. In addition, fewer endometrial glands were observed in *WNT4* knockout mice [73]. The present findings reinforce the involvement of *WNT7B* in estradiol‐stimulated endometrial gland development. However, the study also suggests that *WNT7B* may not be the sole factor for regulation of endometrial gland development, indicating that other mediators (such the factors mentioned above), could also play a part.

Beyond the Wnt pathway, this study showed an extrinsic regulation through HESC‐derived TGFβ1 interaction with VDR during endometrial gland development. TGFβ1‐VDR signaling regulates the Wnt/β‐catenin pathway, and inhibits cell migration by reducing β‐catenin expression in vitro [78, 79]. Sustained TGFβ signaling increases expression of Wnt ligands, including Wnt4, Wnt7a, Wnt11, and Wnt16, and impairs endometrial gland development in mouse uteri [80]. TGFβ1 also plays a role in endometrial repair in menstrual cycles [81] with expression in the endometrium peaking just after the menses and gradually decreasing throughout the proliferative phase when significant regeneration of the endometrial glands occurs [82]. The observation is supported by reduced TGFβ1 expression in the human endometrium from the proliferative to the secretory phases [83]. As the interaction between TGFβ1 and Wnt signaling pathway is complicated especially in human reproductive system, alternative regulatory mechanism besides TGFβ1‐VDR interaction is also possible, which may need deeper investigation in the future.

The hormone‐dependent nature of endometrial tissue introduces an additional regulatory mechanism suppressing *WNT7B* levels and promoting endometrial gland development through estradiol signaling. The finding aligns with the physiological role of estradiol in the menstrual cycle, particularly in the proliferative phase, when the increasing estradiol levels support the growth and elongation of endometrial glands in preparation for implantation [1]. The in vitro observations were validated using an in vivo mouse model. Unlike humans, mice have an estrous cycle lasting 4‐5 days, comprising the proestrus, estrus, metestrus, and diestrus stages [84]. To focus on endometrial gland development, estradiol was administered to mice in the proestrus stage. The observed reduced WNT7B expression in the estradiol‐stimulated endometrial glands in mice corroborates the in vitro findings that estradiol induced *WNT7B* downregulation and promoted assembloid‐derived tubular gland formation.

Translating findings from laboratory models to clinical observations strengthens the physiological relevance of the proposed mechanisms. During IVF treatment, exogenous gonadotrophin injections during controlled ovarian stimulation enhanced growth of multiple ovarian follicles. Thus, IVF patients had higher serum estradiol concentrations compared to women in a natural cycle at the same phase of the menstrual cycle. The observation that they had significantly lower WNT7B expression in the endometrial glands is consistent with the in vitro and in vivo results, emphasizing the importance of *WNT7B* downregulation in response to estradiol. Another study found significantly higher expression of Dickkopf homolog 1 (DKK1), a known Wnt signaling inhibitor, in the endometrium of women undergoing controlled ovarian stimulation compared to women with regular cycles [85], which also aligns with the observation of in this study.

As with any model system, recognizing limitations provides direction for future improvements and research opportunities. One limitation of this study is the inability to isolate assembloid‐derived tubular gland structures from the collagen‐Matrigel‐hydrogel for sustained growth and downstream analysis. While sorting the *EPCAM*‐positive glandular cells could potentially address this issue, the loss of cell‐cell interactions and 3D structure during cell sorting remains a significant obstacle. Another limitation of our study is the reliance on 2D projections to quantify tube formation. Future studies will employ volumetric 3D reconstructions to more accurately measure gland volume and branching topology, enabling a direct assessment of spatial self‐organization. Additionally, our assembloids did not include other important cell types, such as immune and endothelial cells, which play crucial roles in the human endometrium. Since our research focused on epithelial‐stromal crosstalk, the model does not capture the full complexity of the in vivo endometrium. Future efforts should consider incorporating a microfluidic system to create a more comprehensive in vitro model of the endometrium [16]. In vivo, our estrogen‐stimulation experiment used intact cycling mice with PBS vehicle as a physiological baseline rather than ovariectomized, hormone‐replaced, phase‐matched comparators. Future work will employ ovariectomized with controlled hormone replacement and terminal phase matching to isolate estradiol‐specific effects on WNT7B and gland morphogenesis. Finally, we have not yet conducted comparative validation against primary human endometrium or in vivo repair models. Future studies will address this by benchmarking against phase‐matched patient tissues using histology, multiplex immunofluorescence, and spatial transcriptomics and by employing tractable mouse repair models, including hormone‐withdrawal (“menses‐like”) and intrauterine injury paradigms. In addition, we will utilize human endometrial xenografts to further validate physiological relevance.

To summarize, this study developed a 3D endometrial assembloid model that demonstrates endometrial gland development. Reduced expression of WNT7B is an intrinsic regulatory factor of tubular gland development in EO, which is extrinsically regulated by HESCs through TGFβ1‐VDR interaction. The model bridges the gap between in vitro cultures and the complex in vivo environment, providing a valuable tool for studying glandular biology and a foundation for advanced 3D models of the human endometrium. Our findings also identify estradiol‐stimulated *WNT7B* as a key regulator of endometrial gland formation, which could have significant implications for developing new diagnostic and therapeutic strategies on endometrial disorders, such as endometrial adenomyosis, endometriosis, infertility, and endometrial hyperplasia.

## Author Contributions

The experimental work in this manuscript was conducted by L.X., L.J., G.Y., L.Y., H.K.M.L., and L.L. Manuscript writing was conducted by L.X., L.C.L., and C.P.C.N. Reviewing was conducted by Z.Q., L.K.F., C.K.W., N.E.H.Y., Y.W.S.B., L.C.L. and C.P.C.N. Project supervision was conducted by L.C.L and C.P.C.N.

## Funding

This work was supported by Hong Kong Research Grant Council General Research Fund (17110423 & 17114424) and PolyU (UGC) Start‐up Fund for New Recruits (P0050668).

## Ethics Statement

The animal experiments were approved by the Committee on the Use of Live Animals in Teaching and Research (CULATR: 5317‐20; 5938‐21) of The University of Hong Kong.

## Conflicts of Interest

The authors declare no conflicts of interest.

## Supporting information




**Supporting File 1**: advs73419‐sup‐0001‐SuppMat.docx.

**Supporting File 2**: advs73419‐sup‐0002‐Data.xlsx.

## Data Availability

All data are available in the main text or the supplementary materials. All bioinformatic analyses were performed using publicly available software as described in Materials and Methods. The single‐cell RNA seq and mRNAseq data were deposited in the NCBI GEO database (https://www.ncbi.nlm.nih.gov/geo/), under the accession code GSE292827 and GSE294012.
