# Supplementary material for: Endometrial Assembloid Model Reveals Endometrial Gland Development Regulation by Estradiol‐Driven WNT7B Suppression
Source: Adv Sci (Weinh). 2025 Dec 22;13(13):e09664. doi: 10.1002/advs.202509664 (PMC12955949; doi:10.1002/advs.202509664)
Supplement: Supplementary file 1 — Supporting File 1: advs73419‐sup‐0001‐SuppMat.docx. [file ADVS-13-e09664-s001.docx]

**Supplementary Figure S1. EO viability and tubular gland formation from EOs under EO-only, simple co-culture and assembloid co-culture condition.**

1. Expression of tight junction marker ZO-1 in the assembloid. Red: ZO-1. Blue: DAPI. Scale bar, 50 μm.
2. Representative image for tubular gland measurement from EOs. Scale bar, 100 μm.
3. Schematic illustration for simple co-culture method and EO-only culture method.
4. EO viability in the 3D co-culture model under EO-only, simple co-culture and assembloid co-culture condition. Viability was calculated by the number of live EOs after 7 days of culture in the Collagen-Matrigel-hydrogel. **: P<0.01, ***: P<0.001, ****: P<0.0001. n=4.
5. Representative images for EOs in simple co-culture and EO-only condition in Matrigel and on day 2 and day 10 in the Collagen-Matrigel-hydrogel. Scale bars, 100 μm. All data was presented as mean ± standard deviation. All the results were analyzed by the Kolmogrov-Smirnov normality test. Statistical comparison was conducted using the Student t-test for two groups or one-way ANOVA with multiple comparison for more than three groups of variables.


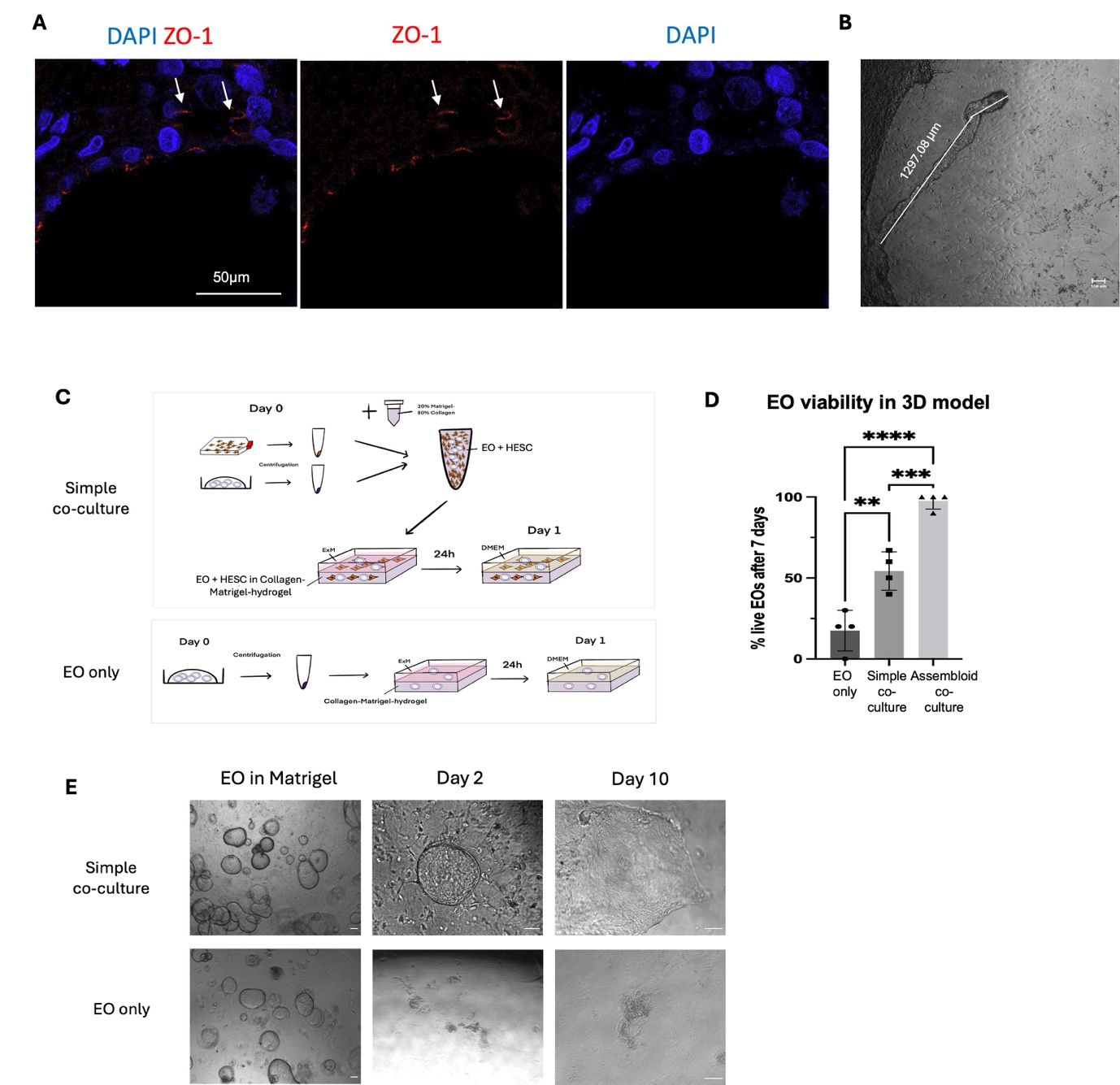


**Supplementary Figure S2. Secretome of assembloid-derived tubular glands and their impact on EVT invasion.**

1. Enriched GO biological function and enriched Kyoto encyclopedia of genes and genomes (KEGG) analysis for DEPs in Day 7 conditioned medium (CM) versus Day 1 CM.
2. Expression levels of four high-ranking DEPs (PAEP, MUC-1, MMP-3 and IL-6) in Day 7 and Day 1 conditioned medium collected from EO/HESC co-culture (n=3) in proteomic analysis result. Day 7 and Day 1 conditioned medium collected from HESC monoculture was used as control group (n=1).
3. Western blot analysis for PAEP, MUC-1, MMP-3 and IL-6 in Day 1 conditioned medium (D1 CM) and Day 7 conditioned medium (D7 CM) with representative blot. Coomassie blue staining was used as protein loading control. *: P<0.05, **: P<0.01, ***: P<0.001. n=4.
4. Immunohistochemical staining for PAEP, MUC-1, MMP-3 and IL-6 in secretory phase human endometrial tissue. Nuclei were counterstained with Haematoxylin. Scale bars, 50 μm.
5. Establishment of EVT from TSC. Representative images of TSC and *in vitro* differentiated EVT cells. RT-qPCR analysis for relative mRNA expression level of *HLA-G*, *MMP2* (EVT markers) and *TEAD4* (TSC marker) in TSC and EVT cells. **: P<0.01, ****: P<0.0001. Scale bars, 100 μm. n=3. All data was presented as mean ± standard deviation. All the results were analyzed by the Kolmogrov-Smirnov normality test. Statistical comparison was conducted using the Student t-test for two groups or one-way ANOVA with multiple comparison for more than three groups of variables.


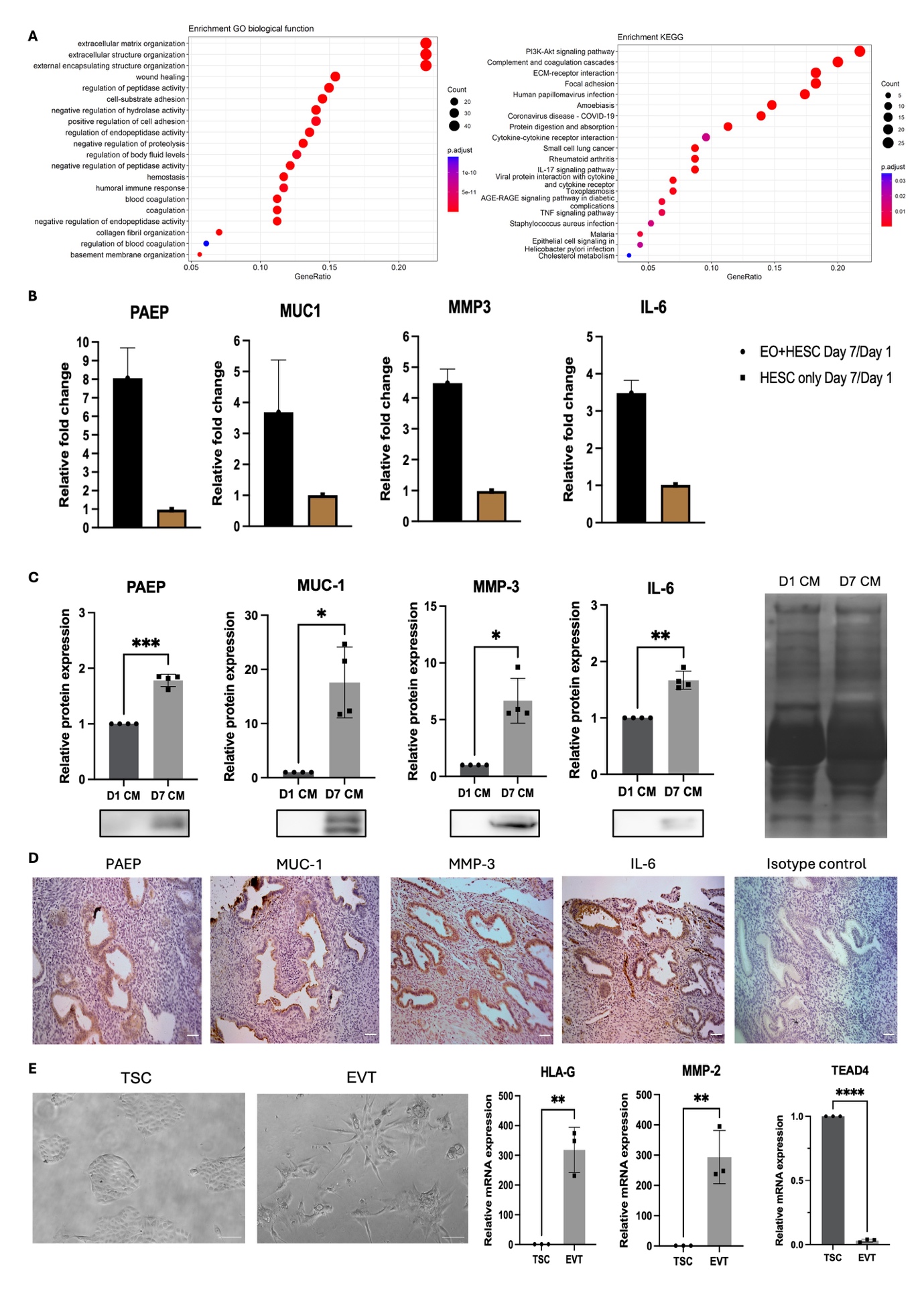


**Supplementary Figure S3. Comparison of single-cell RNA sequencing result from human full-thickness endometrial tissue with 3D EO/HESC co-culture model.**

UMAP plot for single-cell RNA sequencing analysis of full-thickness human endometrium and day 1/day 9 EO/HESC co-culture model. eS: endometrial stromal cells; Glandular epithelia: endometrial glandular epithelial cells.


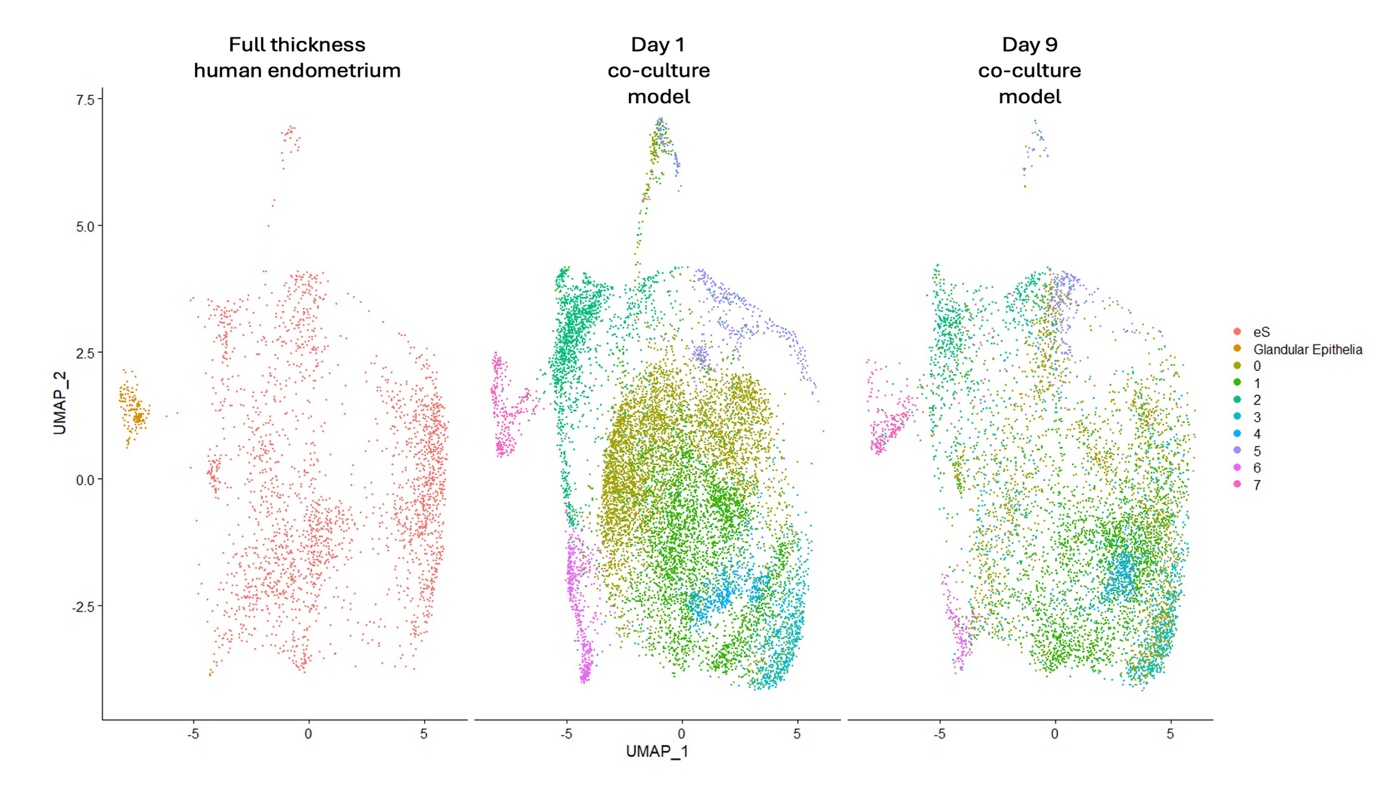


**Supplementary Figure S4. DEG identification and validation from sequencing analysis result.**

1. Shortlisted DEGs according to single-cell RNA sequencing and bulk RNA sequencing with expression tendencies and fold change (FC).
2. RT-qPCR analysis for relative mRNA expression level of *WNT7B*, *KCTD12*, *RGS22*, *LCN2*, *LRRC56* and *SLC22A15* in EOs with and without tubular gland development capacity. ****: P<0.0001. n=4. All data was presented as mean ± standard deviation. All the results were analyzed by the Kolmogrov-Smirnov normality test. Statistical comparison was conducted using the Student t-test for two groups or one-way ANOVA with multiple comparison for more than three groups of variables.


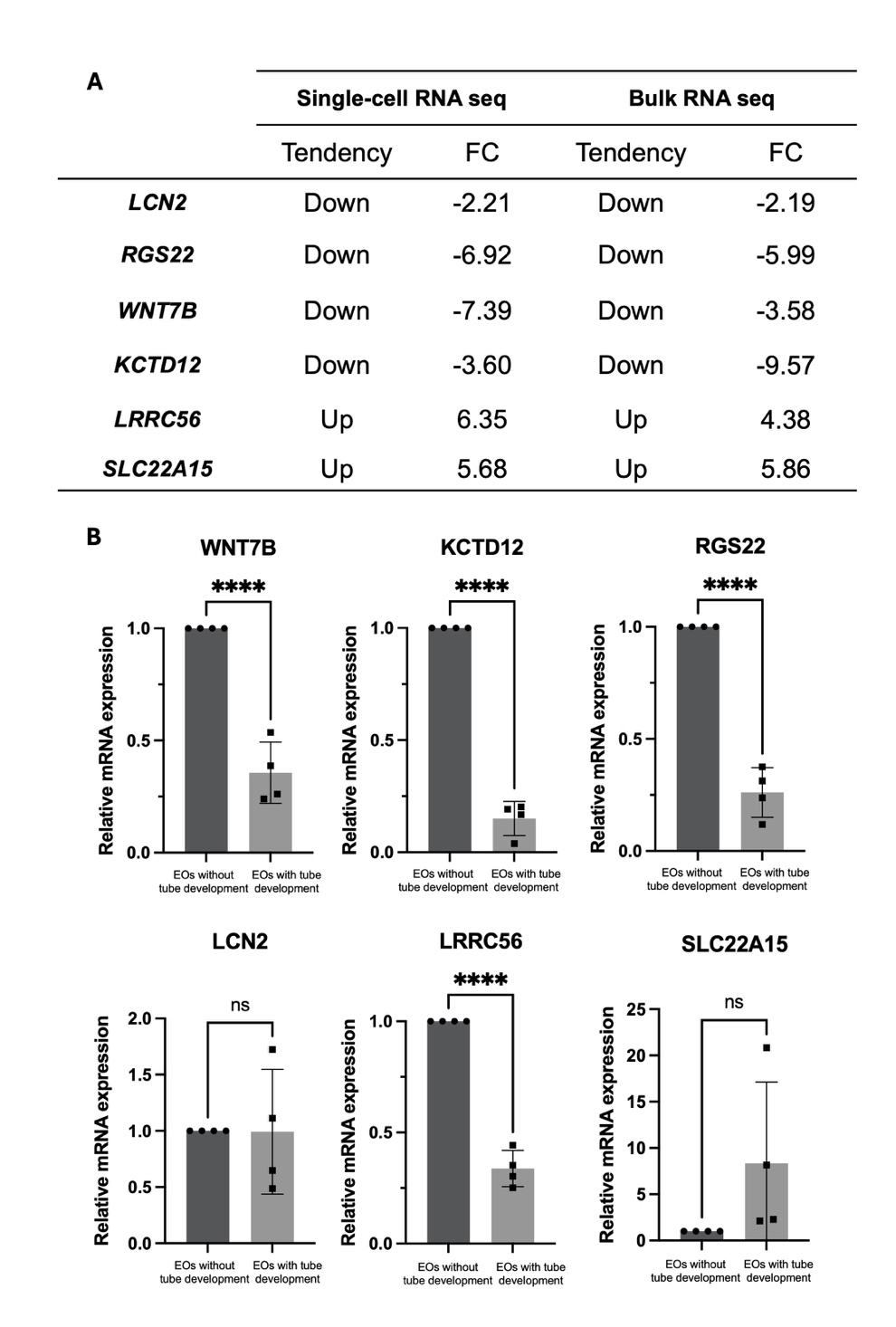


**Supplementary Figure S5. VDR-TGFβ1 interaction between EOs and HESCs.**

1. Co-immunoprecipitation demonstrating VDR–TGFβ1 interaction (full blot). Recombinant VDR or EO cell lysate was incubated with recombinant TGFβ1. VDR-containing complexes were immunoprecipitated using an anti-VDR antibody and Protein G beads. The precipitated complexes were analyzed by Western blotting with anti-TGFβ1 and anti-VDR antibodies. n=3. Figure shows a representative image from three independent experiments.
2. Secretion of TGFβ1 in EO and HESC conditioned medium examined by ELISA. EOM: EO conditioned medium; HESCM: HESC conditioned medium. **: P<0.005. n=4.
3. Expression of WNT7B in EOs after treatment with VDR inhibitors (TEI-9648, TEI-9647 and ZK168281, 1 μM) and TGFβ1 (10 ng/ml) for 48 hours. **: P<0.005. n=3.

**
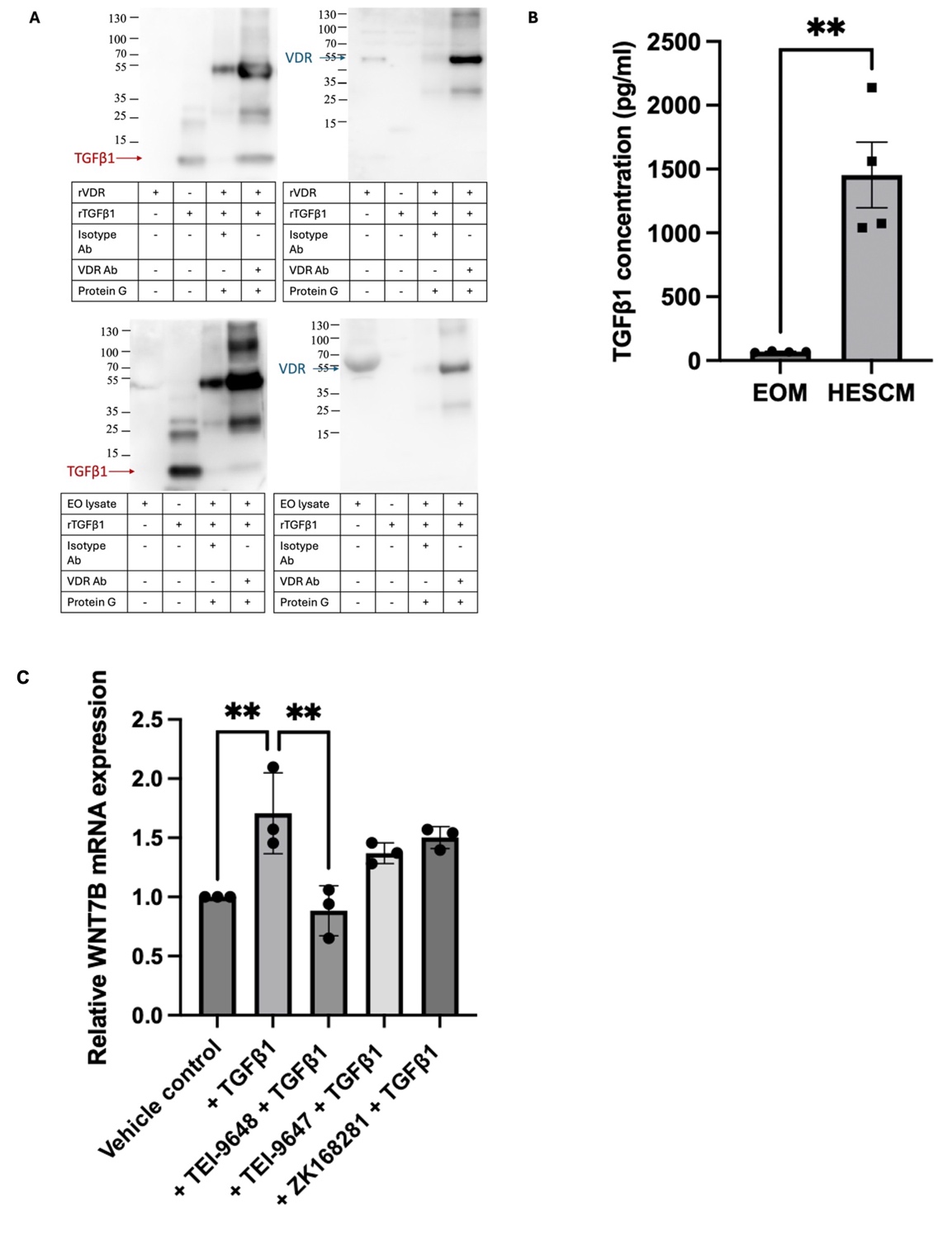
**

**Supplementary Figure S6. Estradiol responsiveness in EOs.**

1. Immunohistochemical staining for ESR1 in wildtype EOs (control) and EOs treated with 10 nM/100 nM of estradiol (E2 10 nM; E2 100 nM) for 7 days. Nuclei were counterstained with Haematoxylin.
2. Average proportion of ESR1 positive cells per EO. Scale bars, 100 μm. ****: P<0.0001. n=5. Proportion of ESR1+ cell per EO was calculated by the number of cells with positive ESR1 signal divided by the number of nuclei counterstained with Haematoxylin. All data was presented as mean ± standard deviation. All the results were analyzed by the Kolmogrov-Smirnov normality test. Statistical comparison was conducted using the Student t-test for two groups or one-way ANOVA with multiple comparison for more than three groups of variables.


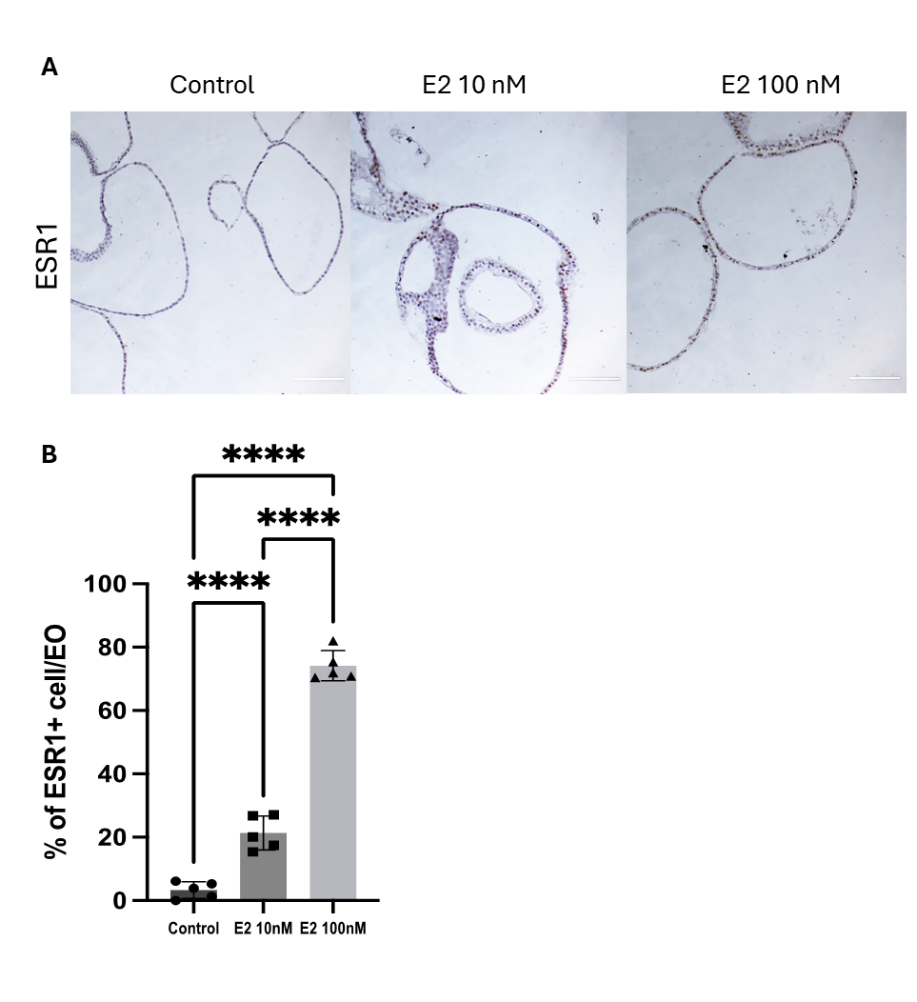


**Supplementary Figure S7. Establishment of estradiol-stimulated mouse model.**

1. Vaginal smear staining at proestrus phase. Nuclei were counterstained with Haematoxylin. Scale bar, 100 μm.
2. Uterus of mouse with i.p. PBS injection (control) or 100 μg/kg of estradiol injection (E2) for 14 days. All data was presented as mean ± standard deviation. All the results were analyzed by the Kolmogrov-Smirnov normality test. Statistical comparison was conducted using the Student t-test for two groups or one-way ANOVA with multiple comparison for more than three groups of variables.
3. Mean uterine mass-to-body weight ratio in PBS/E2 injected mice. ***: P<0.001. n=3.
4. Average endometrial gland number and diameter in PBS/E2 injected mice. *: P<0.05; ***: P<0.001. n=3.


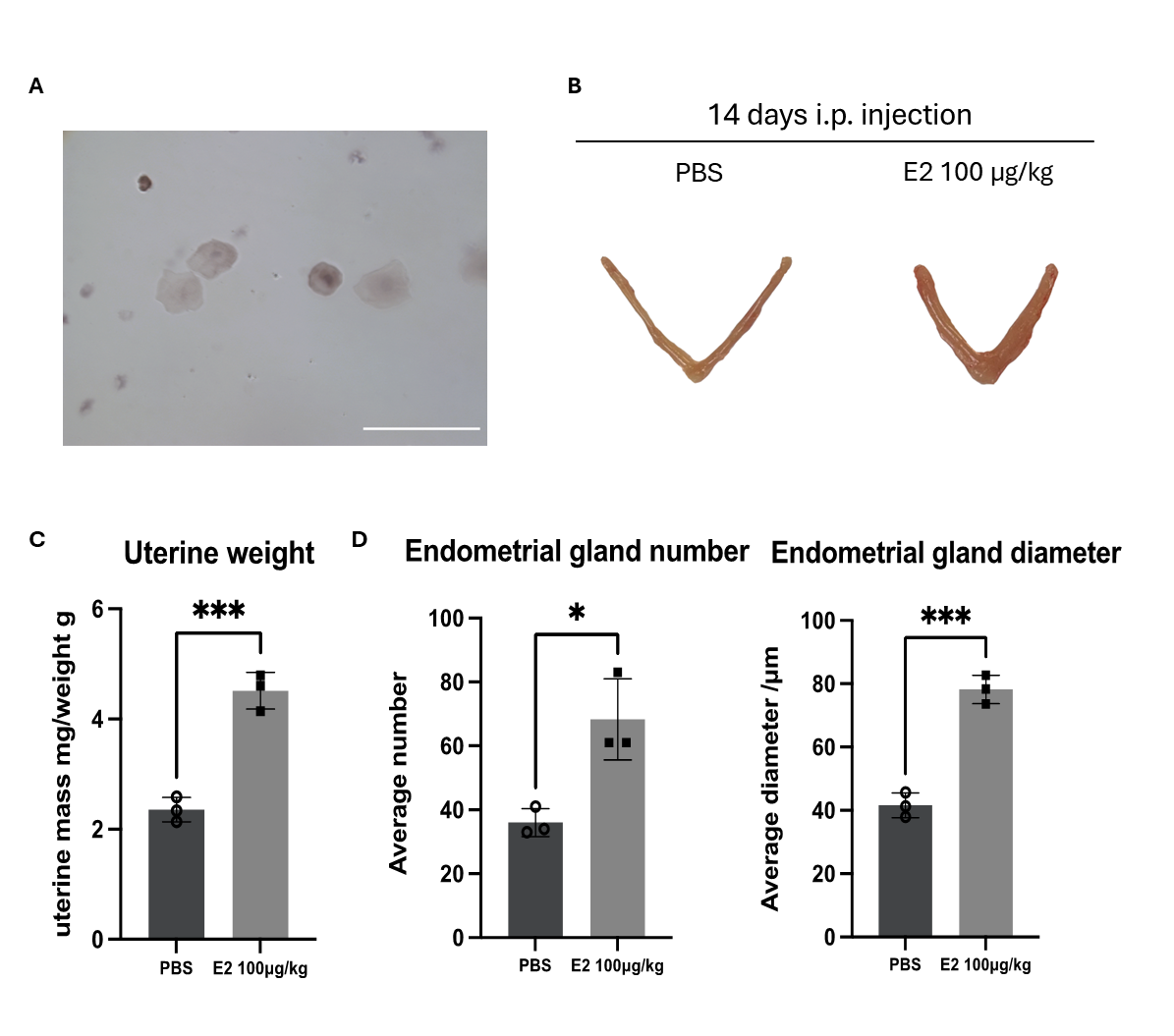


**Supplementary Figure S8. Expression of TGFβ1 in endometrium of women undergoing controlled ovarian stimulation.**

1. Immunohistochemical staining for TGFβ1 in endometrial tissue of female participants with natural menstrual cycle and controlled ovarian stimulation. Nuclei were counterstained with Haematoxylin. Scale bars, 100 μm.
2. Quantification for IHC staining result. *: P<0.05. n=3.


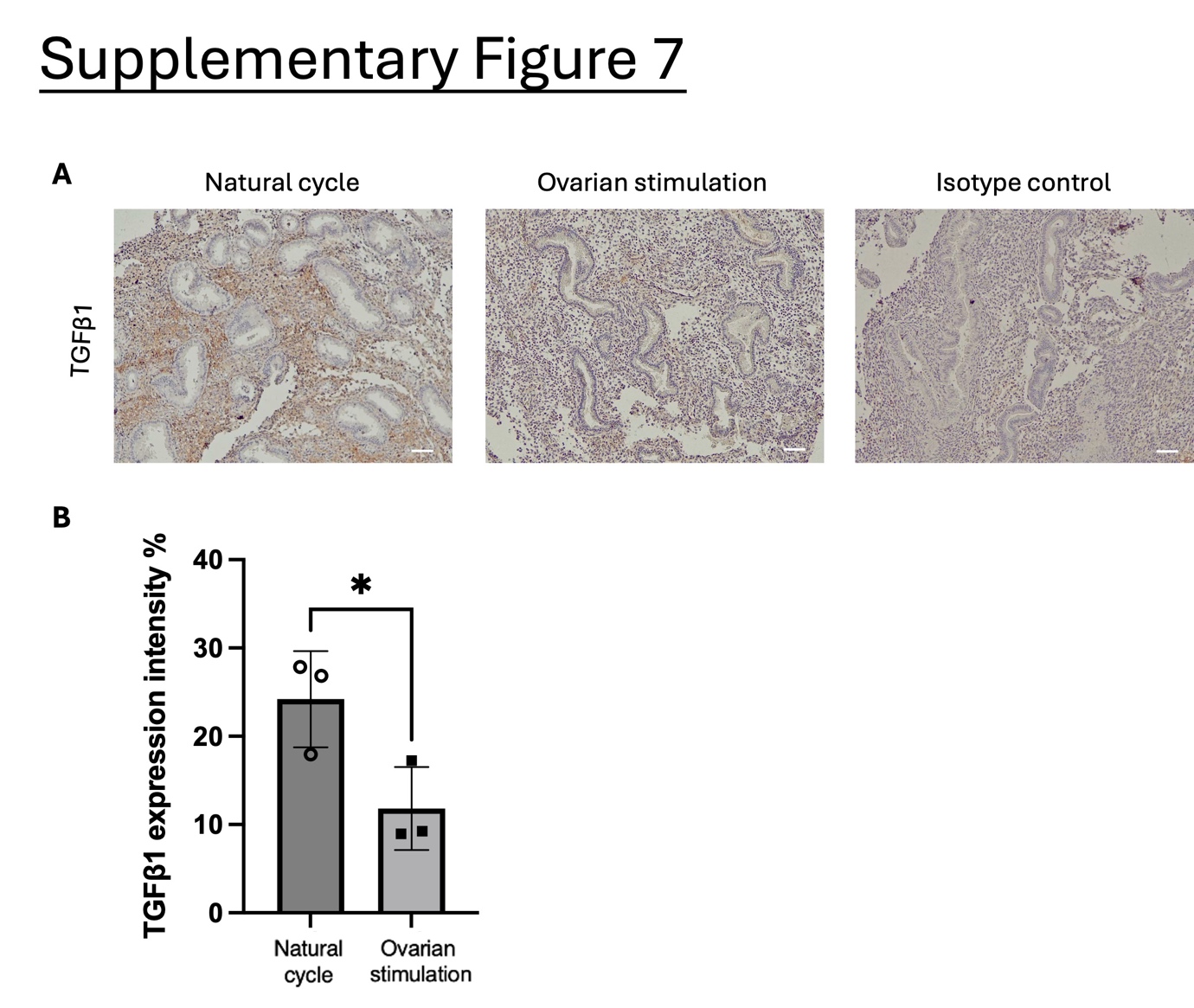


**
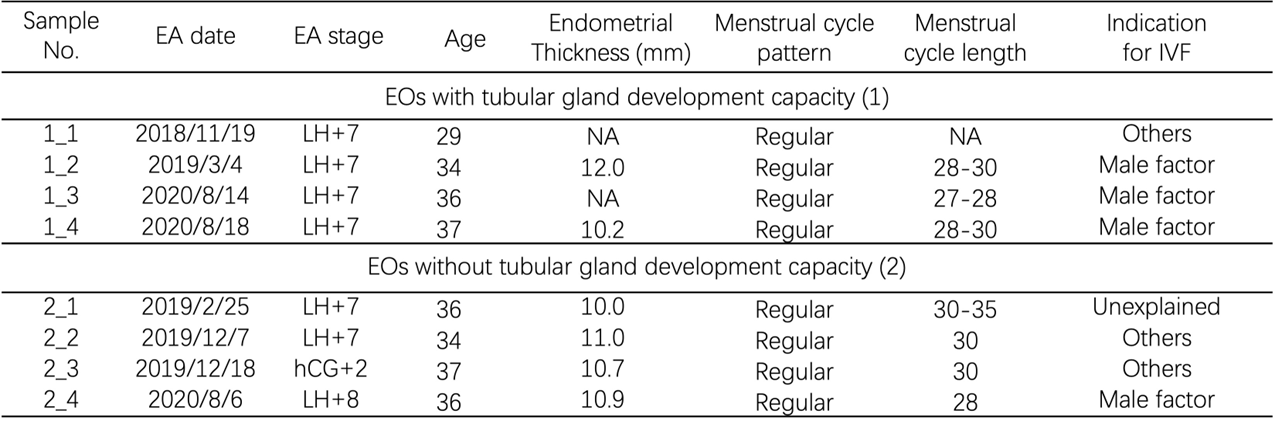
**

**Supplementary Table S1. Demographic information of endometrial tissue donors for EOs with and without tube formation.**

Endometrial aspiration (EA) date, stage, age, endometrial thickness, menstrual cycle pattern, menstrual cycle length and female indication for IVF in EOs with (1) and without (2) tubular gland development capacity. Data was obtained from n=4 women in each group.
